# A Guide to Human Zinc Absorption: General Overview and Recent Advances of In Vitro Intestinal Models

**DOI:** 10.3390/nu12030762

**Published:** 2020-03-13

**Authors:** Maria Maares, Hajo Haase

**Affiliations:** 1 Technische Universität Berlin, Chair of Food Chemistry and Toxicology, Straße des 17. Juni 135, 10623 Berlin, Germany; 2 TraceAge—DFG Research Unit on Interactions of Essential Trace Elements in Healthy and Diseased Elderly, D-13353 Potsdam-Berlin-Jena, Germany

**Keywords:** zinc, intestinal absorption, zinc homeostasis, zinc bioavailability, zinc uptake, in vitro intestinal model, Caco-2, intestinal

## Abstract

Zinc absorption in the small intestine is one of the main mechanisms regulating the systemic homeostasis of this essential trace element. This review summarizes the key aspects of human zinc homeostasis and distribution. In particular, current knowledge on human intestinal zinc absorption and the influence of diet-derived factors on bioaccessibility and bioavailability as well as intrinsic luminal and basolateral factors with an impact on zinc uptake are discussed. Their investigation is increasingly performed using in vitro cellular intestinal models, which are continually being refined and keep gaining importance for studying zinc uptake and transport via the human intestinal epithelium. The vast majority of these models is based on the human intestinal cell line Caco-2 in combination with other relevant components of the intestinal epithelium, such as mucin-secreting goblet cells and in vitro digestion models, and applying improved compositions of apical and basolateral media to mimic the in vivo situation as closely as possible. Particular emphasis is placed on summarizing previous applications as well as key results of these models, comparing their results to data obtained in humans, and discussing their advantages and limitations.

## 1. Introduction

The essential trace element zinc plays a key role for several important biological processes in the human body [1]. To compensate the endogenous zinc loss and maintain a healthy zinc homeostasis, this micronutrient has to be supplied with food on a daily basis [2]. Human body zinc homeostasis is predominantly regulated by its intestinal absorption [3]. In this process, zinc transporters on the apical and basolateral membrane of enterocytes are engaged and regulate cellular and body zinc homeostasis together with the cellular zinc-binding protein metallothionein [4,5]. Despite this knowledge and ongoing research, a deeper understanding of the molecular processes regulating zinc absorption via the intestinal epithelium is still scarce. Zinc absorption does not only depend on an adequate dietary intake, but is also greatly affected by its intestinal availability from the diet. To further illuminate the impact of these factors on zinc absorption by the intestinal epithelium remains one of the determining topics in research [6,7]. Herein, in vivo human studies using (stable) isotope techniques are still the gold standard [8]. During the past 50 years, attempts to establish suitable three-dimensional in vitro cell culture models to mimic in vivo processes have gained more attention. This is mainly due to high costs and ethical standards of animal studies and the benefits of in vitro models providing a microenvironment that advances studies of cellular processes on a molecular level [9,10].

This review aims to provide an overview of the current knowledge on human intestinal zinc absorption, including the major cellular processes and nutritional aspects. In addition, the scope of this review is to illustrate analytical approaches that have been applied to characterize human zinc absorption, particularly the achievements and advantages of in vitro cellular intestinal models to investigate molecular regulatory parameters and transport kinetics of human zinc absorption as well as the bioavailability of this micronutrient from food.

## 2. Zinc Homeostasis and Its Role in Human Health

**Figure 1:**
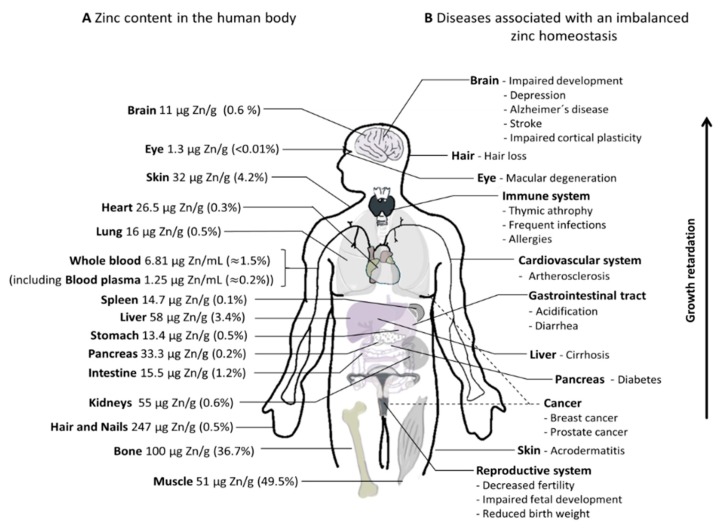
Overview of zinc distribution and disease association in the human body. (**A**) Approximate zinc content (µg per g wet weight) of the respective tissues and the resulting proportion of total body zinc. Detailed estimation of the tissues’ zinc content and references are depicted in Supplementary Table S1. (**B**) Diseases of the respective organ systems associated with imbalanced zinc homeostasis.

Zinc is the second most abundant micronutrient in the human body after iron [11,12]. Based on bioinformatics research, approximately 2800 human proteins are presumed to bind zinc [13], potentially requiring the divalent cation for catalytic, structural, and regulatory functions [5,13]. Hereby, zinc is crucial for gene expression, is needed for the activity of several metalloenzymes, and provides a major structural component in zinc fingers and zinc finger-containing domains [14,15]. Consequently, zinc is essential for various cellular processes such as differentiation, apoptosis, and proliferation, which influences growth and development of an organism [16]. Moreover, in the past two decades, the knowledge about its importance as a signaling molecule increased [17], particularly in the immune system and as a neuro-modulator in synaptic vesicles [18]. To fulfill this multiplicity of functions, zinc needs to be properly distributed into all compartments of the human body, and the differences in zinc content between various organs (Figure 1, left hand side, calculation and references in Supplementary Table S1) highlights the existence of a complex homeostasis ensuring proper allocation. The adult human body contains approximately 2.6 g of zinc. The largest fraction is localized in bone and skeletal muscle (~86%), followed by skin (4.2%), and liver (3.4%). It has to be noted that some zinc-containing entities, such as the thymus and mucous membranes, are not included in the calculation, so the actual total zinc content of the human body is higher than the 2.6 g estimated in Figure 1. Moreover, according to Jackson et al., validity of such calculations is limited since they are mostly based on zinc content from biopsies and sections of tissues assuming that this reflects the total zinc content of the respective live tissue [19].

Plasma or serum zinc levels in healthy individuals vary from 12 to 16 µM [20,21,22], which corresponds to less than 1% of whole-body zinc. It is mainly bound to albumin (60%), α-macroglobulin (30%), and transferrin (10%) [23], which leaves only a sub-nanomolar concentration of free zinc [24,25,26,27]. Still, serum represents a rapidly exchangeable zinc pool of high importance for distributing zinc within the body. In contrast, skeletal muscle and bone comprise zinc with a lower turnover and slower availability for systemic zinc homoeostasis [28].

The importance of zinc homeostasis is highlighted by the remarkable number of diseases associated with alterations in tissue zinc levels, which is summarized in Figure 1 (right hand side). As there is no dedicated compartment for zinc storage in the human body, zinc has to be continuously replenished by dietary intake [2], replacing intestinal and non-intestinal losses of endogenous zinc [16,29]. Based on several human studies, these losses include fecal zinc excretions and excretions with urine, sweat, menstrual flow, and semen (for adults) as well as loss of hair, nails, and desquamated skin [30]. To this end, currently, human requirements are mostly estimated using a factorial approach considering the overall zinc losses including additional physiological requirements during pregnancies, lactation, or early infancy, as well as the bioavailability of the mineral from the diet [29,30]. Table S2 depicts daily recommendations for dietary zinc intake from different governmental agencies and non-governmental organizations.

The main regulatory mechanisms for human zinc homeostasis are absorption and excretion [3], and the small intestine, pancreas, and liver play central roles in its maintenance [3]. Endogenous zinc is continuously excreted into the intestinal lumen, from which parts are reabsorbed [3], while the remainder, varying between 0.8 and 2.7 mg zinc/d, is excreted with feces [31,32,33,34]. Thus, the close interplay of absorption of exogenous zinc as well as the excretion and reabsorption of endogenous zinc provides a stable balance of body zinc homeostasis. The latter is maintained over a wide range of exogenous zinc intakes [3,35,36,37,38]. In zinc-deficient states, fecal and urinal zinc losses are rapidly decreasing [31,39,40]. Only when these mechanisms fail to sustain zinc-requiring processes, plasma zinc declines [35,40]. This is followed by a reduction of the less exchangeable zinc from tissues such as liver, testes, and bones [5,41]. Consequently, the plasma zinc level itself is not a reliable biomarker for body zinc status [5,27], especially since it also changes during inflammation [42] in response to stress or even after a meal [5].

Inadequacy of the zinc status can be connected to insufficient food supply, but mostly results from poor bioavailability from the consumed diet [36]. Zinc deficiency has high prevalence in developing or poor countries [43,44]. Yet, vegans [45], vegetarians [45], elderly [30,44], and people with disorders connected to a diminished zinc absorption, such as *acrodermatitis enteropathica* or celiac disease [46] as well as diseases that cause increased zinc loss, such as inflammatory bowel diseases [47,48], are also susceptible.

According to the World Health Organization (WHO), one-third of the world’s population are at risk for zinc deficiency [49]. The lack of a suitable biomarker for physiological zinc status, and, thus, a low possibility to recognize insufficient zinc absorption, particularly in the early stages of a mild zinc deficiency, is a major obstacle in this situation [16,50]. An imbalanced zinc status or deficiency of this micronutrient is associated with severe health consequences (Figure 1), which causes high morbidity. Zinc excess, on the other hand, is mainly associated with disturbed copper homeostasis (reviewed in detail in Reference [16]). Symptoms of zinc deficiency are reversible [16,51,52,53]. In most cases, zinc supplementation in addition to dietary zinc provides a convenient option to compensate for inadequate zinc intake, malabsorption, or increased zinc loss due to intestinal diseases [54,55,56].

## 3. Zinc Absorption

Zinc is absorbed throughout the whole small intestine [57,58], but the major site of intestinal zinc absorption in humans remains controversial. In rats, the highest absorption rate is reported either in the duodenum and ileum [59,60,61], or only in the ileum [62] or jejunum [58,63], respectively. In vivo studies investigating the actual site of zinc absorption in humans are scarce. However, using small intestine perfusion techniques in healthy individuals, the major absorption sites in human intestines are found to be both the duodenum [64] and jejunum [58].

Zinc uptake takes place at the intestinal brush border membrane, where it is transported from the lumen into absorptive cells of the epithelium: the enterocytes. The subsequent excretion of the cation at the basolateral side of enterocytes releases it into the portal blood, where it is predominantly bound to albumin, which distributes the metal in the body [3,65]. While several in vitro studies show transport from the basolateral to the luminal site of the intestinal epithelium [66,67,68], this has not been observed in humans so far [69]. Additionally, only a rather low apical zinc secretion into the lumen is reported in vivo using perfused rat intestines and physiological serum zinc concentrations [70].

Zinc absorption kinetics are described by carrier-mediated and saturable processes [58,69,71,72], whereby zinc uptake at the apical membrane of the intestinal mucosa seems to be the rate limiting step [70]. Saturation of these transport mechanisms at a certain luminal zinc level is reflected by an absorption plateau with a half saturation constant (K_m_) of cellular zinc uptake in vivo of 29–55 µM zinc [38,70,73]. However, at higher luminal zinc concentrations, zinc uptake becomes non-saturable, which indicates passive diffusion [3,57,71]. Notably, the ‘high zinc concentrations’ applied in these studies varied from >200–1000 µM [57,71,72]. This might not be relevant in vivo for normal zinc intake, as physiologically relevant concentrations in the intestinal lumen after consumption of a standard meal vary around 100 µM [58,64,74] for which a saturable and carrier-mediated transport kinetic applies both in in vitro and in vivo studies.

Fractional absorption of dietary zinc in humans is typically in the range of 16–50% [7,30,33,75,76,77], which is inversely related to oral zinc intake [36]. Moreover, net absorption is regulated by body zinc homeostasis and, thus, depend on the individual zinc status adapting to prolonged low zinc diets. Consequently, zinc-deficient humans and animals show increased fractional zinc absorption [34,78,79,80], absorbing up to 92% of dietary zinc [34,80]. Accordingly, human zinc absorption is more efficient from low zinc diets [7]. Zinc absorption is also affected by the form in which it is administered. Net absorption is higher from orally administered aqueous zinc solutions than the absorption of the same amount of zinc included in a meal [57], mainly because absorption of the mineral depends on its bioavailability in the intestinal lumen, which will be discussed in detail in Section 4.1.

### 3.1. Intestinal Zinc Transporters

**Figure 2:**
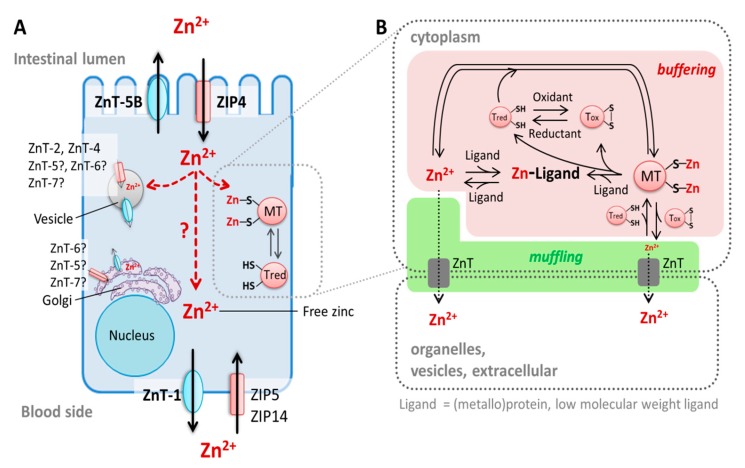
Enterocyte zinc homeostasis. (**A**) Zinc homeostasis in enterocytes during zinc absorption. Three main zinc pools in enterocytes have been described: (i) cytoplasmic-free zinc, which is only complexed by low molecular weight ligands, (ii) protein-bound zinc, depicted here as metallothionein (MT)-bound zinc, and (iii) free zinc stored in vesicles [104]. The vesicular [102,103] and cytoplasmic-free zinc pools [101] are recognized to be involved in zinc absorption by enterocytes [105]. Cellular zinc homeostasis is maintained by three main groups of proteins: the zinc transporter (ZnT)-and the Zrt-, Irt-like protein (ZIP)-family as well as the zinc-binding metallothioneins [99]. They regulate the cytoplasmic-free zinc concentration and provide its distribution into organelles and vesicles. Exporters of zinc from vesicular stores in enterocytes remain to be identified and transfer of the divalent cation through the enterocytes after its uptake by the cells (illustrated by red arrows) is not yet fully understood. (**B**) Zinc buffering and muffling role of metallothioneins (MTs). MTs and other ligands (such as proteins) bind free zinc and, thereby, buffer its cytoplasmic concentration. In addition to zinc transporters, MTs represent zinc muffling moieties, which decrease free zinc content in the cytoplasm by transferring the cation to transporters, sequestering it into organelles, vesicles, or outside the cell. Notably, free zinc itself can also be transported into organelles, whereby, in this process, the ZnT solely undertakes the muffling [100]. Moreover, MTs re-distribute intracellular zinc by transferring it to other ligands, such as metalloproteins [106]. This zinc transfer may be enforced by a redox-active mechanism in which the apo-protein Thionein (Tred) binds the cation, which results in its metal-loaded form, MT, which releases zinc upon its oxidation to Thionin (Tox) (reviewed in Reference [107]).

Intestinal zinc absorption is mainly mediated by the Zrt-, Irt-like protein (ZIP)4 (solute carrier (SLC)39A4), which imports ionic zinc from the lumen into enterocytes [81,82], and ZnT-1 (SLC30A1), which is a basolateral membrane protein exporting zinc on the basolateral side of enterocytes into the portal blood [83] (Figure 2). The basolaterally localized transporters ZIP5 (SLC39A5) and ZIP14 (SLC39A14) complement these two transporters by importing zinc from the blood circulation into enterocytes [84,85]. Moreover, ZnT-5 variant B (SLC30A5B) is localized at the apical membrane of enterocytes [74,86] and functions in a bidirectional manner, transporting both luminal zinc into enterocytes and cellular ions back into the lumen [86,87]. Hence, this suggests that the previously mentioned apical secretion of the mineral could possibly represent an additional regulatory mechanism of cellular and body zinc homeostasis [87,88].

Earlier findings indicated involvement of the divalent metal transporter (DMT)-1, which is a cation transporter of low selectivity, in intestinal zinc uptake [89]. The identification of ZIP4 as the major transporter for zinc uptake and contradictory results in several in vitro studies [90,91,92,93,94], however, challenge the role of DMT-1 in physiological zinc transport.

Even though the exact transport mechanisms of ZIPs and ZnTs are not yet fully elucidated, it is known that these proteins transport ionic zinc [14,95]. Dietary zinc in the intestinal lumen, however, is mainly complexed by food components influencing the actual available and absorbable zinc concentration. In addition to the uptake of the ionic form, zinc is also suggested to be absorbed in complex with certain amino acids by possibly utilizing another transport pathway than ionic zinc [96].

### 3.2. Enterocyte Zinc Homeostasis and Regulation of Intestinal Zinc Absorption

Cellular zinc homeostasis comprises three main zinc pools: zinc bound to proteins, stored in vesicles, and cytoplasmic free zinc (Figure 2A). The latter is only complexed by small molecule ligands [97] and considered to be the biologically active form of the ion [98]. This mobile zinc species is either in transit through the cell, being “re-distributed,” or serves as a signaling molecule [99]. Therefore, the cytoplasmic-free zinc concentration has to be tightly regulated [98] and is buffered to a picomolar level [97], being either transported out of the cell and sequestered into vesicles via ZIP and ZnT transporters, or bound to proteins such as metallothioneins (MTs) [99] (Figure 2A,B). Hence, MTs and zinc transporters represent an elaborate zinc buffering and muffling system [100].

Detailed processes of cellular distribution of zinc into enterocytes and its transfer through the cells after its absorption are not yet completely understood. Examinations of free zinc (pools) in enterocytes in vitro with the eCalwy biosensor [101] and the fluorescent zinc probe Zinpyr-1 [102,103] document that enterocytes contain at least two different free zinc pools that are involved in the maintenance of zinc homeostasis during zinc absorption: cytoplasmic-free zinc and vesicular zinc [101,102,103]. Nevertheless, these processes have to be further scrutinized. In particular, the chronology of the zinc transfer through the enterocytes upon its absorption and its subsequent basolateral release into the blood circulation needs to be unraveled in more detail.

**Figure 3:**
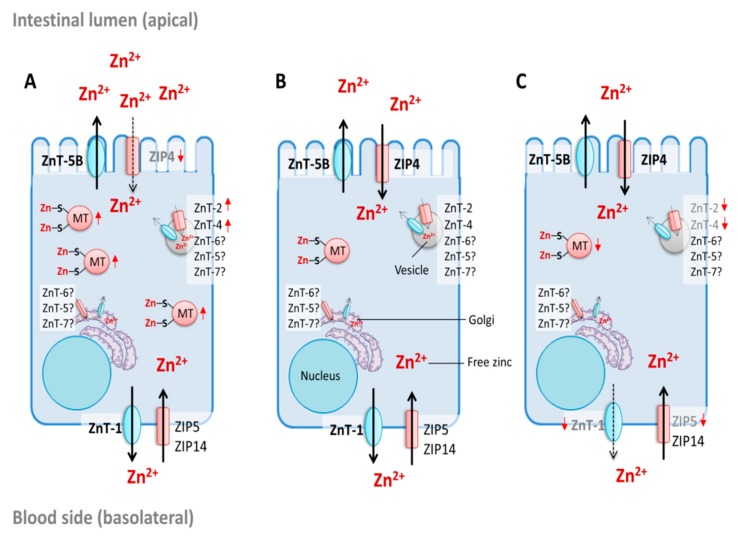
Regulation of intestinal zinc absorption. Potential regulatory mechanisms of zinc absorption into enterocytes during (**A**) zinc excess, (**B**) adequate supply, and (**C**) zinc deficiency, based on experimental data on the zinc-dependent expression pattern of the intestinal zinc transporters (ZnT) and the Zrt-, Irt-like protein (ZIP)-transporters as well as metallothioneins (MT). Enterocyte zinc homeostasis is controlled by these proteins, regulating the amount of intestinally absorbed and basolaterally exported zinc [150]. The subcellular localization of ZnT-5, ZnT-6, and ZnT-7 in enterocytes is not yet fully investigated. Zinc-dependent up-regulation or downregulation of the respective protein and/or messenger ribonucleic acid (mRNA) are indicated by red arrows.

The discovery of intestinal zinc transporters and elucidation of the role of zinc-binding MTs in maintaining enterocyte zinc homeostasis contributed to an increased understanding of regulatory parameters of intestinal zinc absorption. Furthermore, the current knowledge about their regulatory role during this process will be briefly summarized. There are four known MT genes (MT-1–MT-4) encoding eleven functional human MT-isoforms [108,109]. In the intestine, mainly MT-1 and MT-2 are expressed [109]. The singular form “MT” refers to both MT-isoforms for the sake of convenience and readability. Similar to its role in cellular zinc homeostasis in general, MT plays an important role in regulating enterocyte zinc homeostasis by binding zinc that is absorbed into the cells [70]. Thus, the protein controls free levels of the cation and is discussed to mediate zinc trafficking through the cell as well as its transfer to other proteins such as zinc transporters (Figure 3) [108,110]. Hence, MT’s zinc buffering and muffling properties might regulate the amount of zinc that is exported into the portal blood and distributed in the body.

Expression of MT is related to changes in enterocyte zinc levels. Elevated cellular free zinc itself induces *mt* expression via the metal regulatory transcription factor 1 (MTF-1) [111]. Protein and messenger ribonucleic acid (mRNA) levels of intestinal MT increase the response to elevated dietary zinc in animals and humans in vivo [86,112,113,114], acting as an initial defense mechanism against high luminal zinc concentrations [114], whereas, in zinc-deficient states, MT protein and mRNA are decreasing [86,112,113,114]. Furthermore, MT upregulation appears to affect zinc transport kinetics and decreases luminal zinc absorption [71,115,116,117], which leads to decreased serum and body zinc levels in response to elevated intestinal MT [116,118,119]. In an earlier study, MT was also suggested to be involved in zinc export from enterocytes back into the intestinal lumen [117]. Furthermore, luminal secretion of MT after treatment with physiological zinc concentrations was observed in a three-dimensional in vitro intestinal cell model, indicating that MT might also mediate enterocyte zinc homeostasis by apically sequestering excess zinc [120]. The relevance of MTs for zinc trafficking, however, seems to be limited, as MT knockout mice (for MT-1 and-2 genes) are more sensitive to additional dietary zinc, but still viable and reproductive [118,121]. Furthermore, experimental modeling of MTs as mufflers indicated that they are possibly not the only proteins mediating zinc transfer to transporters [122]. These findings imply that there must be other proteins maintaining zinc trafficking through the cell. Accordingly, Cousins and coworkers proposed the involvement of the cysteine-rich intestinal protein (CRIP) as an additional mediator of enterocyte zinc trafficking, which may compete with MT [123]. Yet, CRIP was later shown to be expressed in nearly all organs and suggested to play a role in the immune response [124]. More likely is the existence of another moiety in zinc muffling and transfer through the cell, possibly similar to metallochaperones involved in enterocyte iron and copper homeostasis [125].

Similar to MT, intestinal zinc transporters are not only required for the maintenance of enterocyte zinc homeostasis, but are also decisive for zinc absorption (Figure 3). The main intestinal zinc importer ZIP4 is essential for zinc absorption. This is demonstrated by the zinc malabsorption disease *acrodermatitis enteropathica*, originating from different mutations in the gene encoding human ZIP4 [81,82,126,127]. ZIP4 is regulated by dietary zinc in a transcriptional, translational, and post-translational manner [128]. Moreover, surface localization of enterocyte ZIP4 is regulated by cytoplasmic zinc [129]. Under zinc deficiency, *zip4* mRNA is stabilized [127,130,131] and the protein accumulates at the apical plasma membrane result in elevated zinc uptake [129,132]. Zinc repletion results in endocytosis of the protein [129], and ubiquitin-mediated degradation at even higher zinc concentrations [133,134], while transcription remains unaltered [86].

In contrast to ZIP4, *zip5* mRNA abundance is independent from dietary zinc, whereas its translation is zinc-dependent [130]. During zinc insufficiency, its mRNA remains associated with polysomes without being translated, while the basolateral plasma membrane protein is internalized, which minimizes the secretion of body zinc from the blood into the intestinal tract [130,131]. Due to this polysomal stalling mechanism, the protein is again rapidly expressed and accumulates at the membrane after zinc repletion [130]. Consequently, ZIP5 is important for the control of systemic zinc homeostasis and is considered to be involved in sensing the zinc body status [135]. Regulation of *zip14* mRNA, on the other hand, was not altered during dietary zinc deficiency or excess in mice [136].

*Znt-1* mRNA expression is zinc-dependent and, similar to MT, regulated by MTF-1 [83,137]. MTF-1 directly senses cytoplasmic zinc concentrations in enterocytes and regulates ZnT-1 expression, which ensures sufficient capacities for export of the cation into the portal blood and controls intracellular zinc-free levels [108]. Furthermore, aside of its transcriptional regulation, the zinc-regulated surface accumulation of ZnT-1 might also be facilitated on a post-transcriptional level, whereas, during zinc deficiency, the protein is degraded via lysosomal and proteasomal pathways [138]. Nevertheless, in vivo data for basolateral ZnT-1 during zinc excess and deficiency are contradictory and scarce regarding its differential expression in humans. In animal studies, high oral zinc doses increase protein [83] and mRNA expression [83,118,137]. Conversely, *znt-1* mRNA and the corresponding protein are downregulated after zinc supplementation in humans in vivo [86]. Zinc restriction, on the other hand, leads to downregulation of mRNA and protein in weanling rats [139] but not in mature rats [137]. In contrast to the previously mentioned MT knockout mice, ZnT-1 knockout mice already die in an early embryonic state [140].

The apically localized bidirectional zinc transporter ZnT-5B is not affected by zinc deficiency, but is downregulated [86] or upregulated [74,87] with elevated cellular zinc availability in in vitro and in vivo studies. This converse regulation indicates a rather complex role in zinc homeostasis and was suggested to be based on both transcriptional repression and stabilization of its mRNA [88]. Aside of its apically located variant B, ZnT-5 is also distributed in cytoplasmic organelles of enterocytes and goblet cells [141] and considered to be essential for zinc homeostasis, as ZnT-5 knockout mice display impaired growth and bone development [142].

In addition to the zinc transporters at the apical and basolateral membranes of enterocytes, there is evidence that ZnT-2, ZnT-4, ZnT-6, and ZnT-7 also regulate the cytoplasmic zinc concentration in enterocytes. ZnT-2 is a vesicular zinc exporter [143] expressed in the human in vitro intestinal cell line Caco-2 [144] as well as in rat [137,145] and mouse intestines [136]. There are two ZnT-2 isoforms expressed in the small intestine with different (sub-)cellular localization. Liuzzi et al. detected a small isoform of ZnT-2 on vesicles close to the apical membrane in enterocytes of lactating rats [145], whereas the larger isoform, also found in secretory vesicles in mammary epithelial cells, seems to be restricted to mouse intestinal Paneth cells [146]. ZnT-4 is expressed in rat [137,145] and murine small intestines [136,141], mainly located in the perinuclear region of murine absorptive epithelial cells [141] and associated with endosomal vesicles predominantly accumulating in the basolateral side of rat enterocytes [145,147]. In response to high zinc intake, both *znt-2* and *znt-4* are upregulated [136,137], which might result in increased sequestration of zinc into vesicles, whereas their mRNA is downregulated after zinc depletion [136]. ZnT-7 and ZnT-6 are also expressed in the small intestine [141,148,149], but appear to be independent of the intracellular zinc concentration since their mRNA abundance does not change in response to low and high zinc diets [136]. Both transporters are detected in the cytoplasm of absorptive epithelia cells. Their subcellular localization in enterocytes, however, remains unknown. However, both transporters are described to be associated with Golgi and vesicular compartments in various cell types [148,149], which indicates that the trans Golgi network could be involved in the cellular zinc transfer through enterocytes [141].

## 4. Zinc in Nutrition and Its Intestinal Bioavailability

### 4.1. Intestinal Zinc Bioavailability

Zinc bioavailability from a mixed or vegetarian diet based on refined cereal grains is estimated to be 26–34%, whereas 18–26% is absorbed from an unrefined cereal-based diet [30]. The actual amount of absorbed zinc not only depends on the zinc content of the consumed diet (for a detailed summary of zinc content of animal and plant-based foods, refer to Reference [30]), but is highly affected by its intestinal zinc bioaccessibility and bioavailability. The term bioaccessibility in this context includes the potentially free and absorbable zinc concentration in the intestinal lumen [151,152]. Bioavailability describes the amount of zinc absorbed by the cells that is subsequently released into the blood and, therefore, available for systemic circulation and body homeostasis [151].

**Figure 4:**
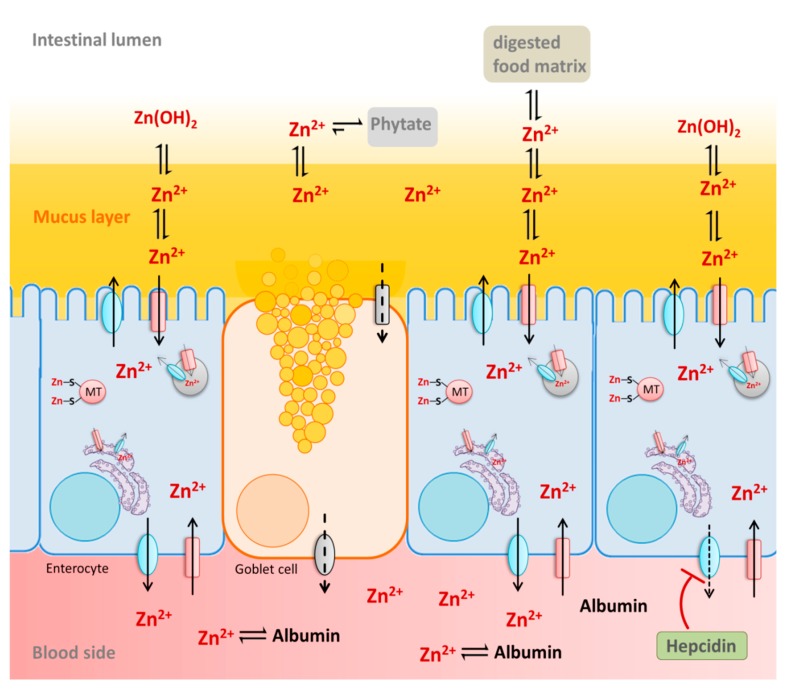
Luminal and serosal factors recognized to influence zinc absorption. Food-derived macromolecules and low molecular weight ligands positively or negatively influence the speciation of the ion as well as its luminal free and available concentration, consequently affecting its absorption by the intestinal epithelium [3]. Phytate forms stable complexes with zinc at intestinal pH, which diminishes its availability for enterocytes [153]. Conversely, the protein content of the consumed food has a positive effect on zinc absorption due to the release of amino acids and peptides upon degradation. Presumably, these increase luminal solubility of the metal, and, consequently, enhance its availability to enterocytes [154,155]. Serum albumin is an important serosal factor, acting as a basolateral zinc-acceptor and enhancing enterocytic zinc release into the blood circulation [102]. Additionally, systemic humoral factors, such as hepcidin, seem to influence ZnT-1-mediated export of zinc by intestinal cells [156], which indicates that the liver might play an important role in secreting humoral factors regulating intestinal zinc absorption.

Due to the digestion process, a wide range of different zinc species is present in the intestine, complexed by food-derived macromolecules or low molecular weight ligands [3]. Hence, zinc accessibility and availability depend on its solubility and stability of the respective complexes in the intestinal lumen. This is affected by the diet as well as by physiological factors such as the mucus layer and the intestinal fluid. Together, these luminal factors alter the speciation of the ion as well as its luminal free and available concentration, which, consequently, affects its absorption by the intestinal epithelium. Below, the beneficial or inhibitory impact of these diet-derived and physiological luminal factors on intestinal zinc bioavailability as well as physiological basolateral factors will be briefly summarized (Figure 4).

### 4.2. Dietary Factors Recognized to Influence Zinc Absorption

Fractional zinc absorption preferentially depends on zinc intake, as its efficiency declines with increased zinc consumption [7,8,32]. Additionally, the zinc species influences its intestinal absorption, which is of particular relevance for zinc supplements (for details refer to Reference [30]).

Phytate, which is a natural component of plants, severely decreases intestinal zinc bioavailability and is regarded as the main nutritional inhibitor of zinc absorption. Notably, the term phytate includes magnesium, calcium, or potassium salts of phytic acid and comprises a mixture of myo-inositol hexaphosphates, pentaphosphates, tetraphosphates, and triphosphates [153]. Actually, tetraphosphates and triphosphates were described to have little impact on zinc absorption, whereas inositol hexaphosphates and pentaphosphates severely impaired intestinal zinc availability in in vivo studies [153,157,158]. Nevertheless, phytate can be hydrolyzed by phytase, which is an enzyme that degrades the molecule to tetraphosphates and triphosphates, consequently increasing zinc availability [159,160]. In contrast to sheep and pigs, which are able to degrade phytate with their own intestinal phytase, levels of this enzyme in human small intestine are very low and, thus, phytate degradation is highly dependent on phytogenic and microbiotic phytase [153,159,161,162]. Phytogenic phytase, particularly in grains, can be activated during fermentation and food processing [159,160,163], which, subsequently, enhances zinc absorption [163].

Zinc is bound by phosphates of phytate, yielding a 2:1 stoichiometry of the zinc/phytate-complex [164] with strong binding affinities: 1.8 × 10^6^ L mol^−1^ (site 1) and 8 × 10^4^ L mol^−1^ (site 2) for myo-inositol hexaphosphate at 37 °C [165]. Moreover, stability of the zinc/phytate-complex is pH-dependent, which illustrates moderate solubility at low pH and poor solubility at pH 7 [165]. Hence, zinc does not even have to be complexed by phytate in the foodstuffs [166] because, at an intestinal pH (luminal pH 6–7.4 [167]), phytate binds the cation effectively, and forms stable complexes with low solubility and bioaccessibility [168,169]. Consequentially, complexed zinc is not available for absorption and is excreted with the feces [170]. Phytate is also discussed to severely impact body zinc homeostasis by binding endogenous zinc that is excreted into the lumen and inhibiting its reabsorption [3]. Thus, the total phytate content of the diet affects the overall zinc bioavailability of a meal. Since the inhibitory effect of phytate on zinc absorption is concentration-dependent, the molar phytate: zinc-ratio of the diet (Table 1) is applied to estimate zinc bioavailability [49] and was shown to be more important than the phytate content of the product itself [171,172]. In general, plant-based diets contain higher phytate levels than mixed diets, which, consequentially, provides less intestinally-available zinc than meat-based diets [45,173].

Significant changes in human zinc absorption are observed starting at a molar phytate: zinc-ratio of 5. Fractional zinc absorption is reduced from 21% in the absence of phytate to 11–16% at a molar ratio of 5–15, and even lower at 4–11% at molar ratios >15 [174]. Additionally, these complexes are stronger in the presence of calcium, which suggests that calcium might aggravate the inhibition of zinc absorption by phytate [174]. However, calcium does not increase the phytate-mediated inhibition of zinc absorption in several human dietary studies [77,166,175]. Other than phytate, fibers such as cellulose seem to have no significant impact on zinc absorption [160,170].

Dietary protein levels positively correlate with zinc uptake [77,154]. Human zinc absorption is substantially higher in the presence of protein from animal sources than plant-based protein [181] and the addition of animal protein to vegetable-based food significantly improved its zinc bioavailability in vivo [182]. This beneficial impact, however, is discussed to be based on the fact that the amount of protein itself counteracts the impairing effect of phytate and not because of its animal origin [183].

Protein is digested in the gastrointestinal tract and degraded into peptides or amino acids [184]. These low molecular weight compounds form complexes with zinc, which increases its bioavailability by enhancing the solubility of the cation in the intestinal lumen [36] and possibly by being absorbed via amino acid transporters [96]. This increases the relevance of zinc complexes with amino acids for zinc supplementation in malabsorption diseases such as *acrodermatitis enteropathica* [96]. Several studies investigated the impact of amino acids on zinc absorption, yielding contradictory results [76,155,185,186,187,188]. Hence, to date, it is not yet feasible to provide a general statement on the effect of amino acids on zinc bioavailability.

The interrelation between different micronutrients and their absorption is still subject to ongoing research. The possible inhibitory impact of calcium on intestinal zinc bioavailability was already discussed above. Furthermore, negative effects of both heme-iron and inorganic iron on zinc absorption were reported by several in vivo studies [185,189,190,191,192], whereby the effect is greater when iron is administered as aqueous solution than together with a meal [185,193]. Copper, on the other hand, has no impact on zinc absorption [194]. In contrast, supra-physiological zinc doses critically impair intestinal copper absorption [195]. Lastly, cadmium [196] and tin inhibit zinc absorption [197]. While the latter study applied unrealistically high amounts of tin, naturally occurring tin concentrations seem to affect zinc homeostasis by increasing its fecal excretion [198].

In contrast to its beneficial role in iron absorption [193], ascorbic acid has no effect on intestinal zinc bioavailability [181,199,200] because zinc, unlike iron, does not need to change its oxidation state for intestinal uptake. Citrate, on the other hand, positively influences zinc availability [201]. Citrate is the main low-molecular weight ligand binding zinc in milk, which, possibly, influences zinc bioavailability from milk and milk products [202]. Concentrations of zinc/citrate-complexes are higher in human milk when compared to cow’s milk [203], which might explain the higher zinc absorption from human milk [204].

Lastly, chemical and physical food processing also affect zinc bioaccessibility and availability [205]. In this context, particularly, the formation of heat-derived zinc-binding ligands, such as Maillard browning products [206,207], decreases its availability, whereas fermentation or germination elevates its accessibility due to phytate reduction [159,208].

### 4.3. Physiological Factors Affecting Zinc Absorption

Aside from dietary components, various physiological factors in the intestinal lumen influence the solubility of zinc and its subsequent availability for the intestinal epithelium. One is the gastrointestinal mucus layer, which enhances the luminal accessibility of the cation and positively influences its bioavailability [69,209]. It is presumed to bind luminal zinc while preventing the formation of insoluble zinc hydroxide [210] as well as hydroxypolymers (Zn(OH)_n_) [211] at intestinal pH of 6–7.4 [167]. Subsequent animal studies confirm this hypothesis and even indicate zinc buffering properties of this physical barrier [211,212,213]. In vitro, gastrointestinal mucins bind zinc with a physiologically relevant affinity, showing a dissociation constant of the mucin/zinc-complex in the same order of magnitude as luminal zinc [103]. Since the mucus layer is not static, but represents a dynamic and viscoelastic gel [214,215], these glycoproteins might assist zinc transport to the underlying epithelium. The ability to bind the cation and buffer free zinc levels that would be available for intestinal cells was studied with human goblet cells and enterocytes [103], and indicates the retention of luminal available zinc [103], corroborating observations from previous animal studies [209,213]. Additionally, a comparison of mucin-producing Caco-2/HT-29-MTX in vitro intestinal model with mucus-lacking Caco-2 monocultures confirmed a beneficial role of mucins for intestinal zinc absorption, which shows enhanced apical zinc uptake and higher fractional absorption when a mucus layer is present [103]. Mucins also bind several other metals, such as iron, lead, calcium, and aluminum [216,217,218] with increasing affinity from M^+^ < M^2+^ < M^3+^ [212]. Consequentially, competitive binding of an ion might influence its luminal availability for the underlying epithelium, while, potentially, explaining the mutual interdependence of intestinal trace element absorption.

Lately, systemic factors were discussed to play a role in intestinal zinc absorption by regulating uptake and transport into the systemic circulation. In this context, Hennigar et al. studied the impact of the liver-derived humoral factor hepcidin, which plays an important role in iron absorption [219], on enterocytic zinc transport [156]. Herein, basolaterally added hepcidin reduces cellular zinc export into the blood by post-translationally downregulating ZnT-1 in the enterocyte cell line Caco-2. Furthermore, the zinc content of enterocytes increases and *mt-1a* is upregulated, which, possibly, controls subcellular zinc pools in enterocytes [156].

Another physiological factor affecting intestinal zinc absorption is the albumin concentration on the serosal side of the intestinal epithelium. Human blood contains about 30–50 mg mL^−1^ human serum albumin (HSA) [220], where it is the main zinc binding and transporting protein [23] and has high zinc binding affinity (molecular ratio of the albumin/zinc-complex 1:2, K_d_ (site A) = 100 nM) [221]. Bovine serum albumin (BSA) acts as a basolateral zinc acceptor, which enhances serosal zinc export on the basolateral side of a three dimensional in vitro Caco-2/HT-19-MTX co-culture model [102]. This remains to be confirmed using HSA, as interspecies differences in the structure of this protein [222] might affect their zinc-binding properties and, consequently, their role as a basolateral zinc acceptor. Notably, in vivo basolaterally applied rat serum albumin enhances fractional zinc absorption in vascular perfusion experiments of rat small intestine, whereas fractional zinc absorption via the intestinal epithelium decreases when no albumin is present [223]. Hence, the presence of albumin in the blood circulation seems to be crucial for the intestinal zinc absorption.

In vitro basolateral zinc excretion of intestinal cells is enhanced by basolateral albumin, whereas cellular zinc uptake from the apical side seems to be unaffected by this zinc acceptor [102]. This also reiterates previous knowledge on intestinal zinc uptake and transport kinetics. The in vivo apical to basolateral zinc transport is a saturable and carrier-mediated process [71], where apical zinc uptake is suggested to be the rate-limiting step [70]. This process is mainly mediated by the apical zinc importer ZIP4 and basolateral zinc exporter ZnT-1 [224], which are both regulated by dietary zinc. Hence, it is rather unlikely that albumin only serves as a thermodynamic sink for the metal in blood and that higher zinc transport in the presence of albumin is only based on a simple diffusion process, following a zinc concentration gradient from the luminal to the basolateral side of the intestinal epithelium. Consequently, this insinuates that albumin might influence the export of zinc from the enterocytes via ZnT-1 into the blood, possibly by interacting with the transporter. However, the underlying regulatory parameters that enhance the basolateral release of zinc in the presence of albumin have to be further investigated.

## 5. In Vitro Studies on Intestinal Zinc Absorption

In the past 50 years, several analytical approaches have been applied to investigate intestinal zinc absorption and its underlying mechanisms. The latter were mainly elucidated with ex vivo animal studies, such as everted rat gut sacs [60,61], Ussing chambers with rat [225,226,227,228,229] and pig [230,231] jejunal segments, and intestinal brush-border membrane vesicles from rat [72] and pig [232,233] small intestines as well as in situ studies with isolated rat intestines using the (vascular) perfusion technique [38,70,115,234] and the intestinal loop method [79]. Moreover, some human studies using perfused intestine were performed as well [58,64]. Conversely, zinc absorption kinetics, fractional absorption, efficiency of transport, and the impact of dietary components on zinc bioavailability were mainly studied in vivo in humans and animals using (stable) isotope techniques [7,32,170,202]. Distinct processes on the cellular level, like the role of zinc transporters and metallothionein, however, were predominantly investigated with in vitro cellular models [74,84,86,129,156], as they provide a standardized and easy platform.

For the three R paradigm of animal testing [235], refined and reduced animal studies can be complemented by in vitro cellular models as vital tools for achieving the “third R” of replacing animal experiments [236]. Moreover, in vitro cellular models provide a standardized microenvironment in which molecular processes can be investigated in detail. Hence, this section will focus on the application of in vitro cellular models in the investigation of intestinal zinc absorption, illustrate aspects to be considered when applying these models, and highlight the advantages of in vitro cellular intestinal models compared to other in vitro or ex vivo methods. The advantages and limitations of these intestinal models for investigating the intestinal zinc absorption are summarized in Table 2. 

### 5.1. Investigation of Zinc Uptake and Transport Using In Vitro Cellular Intestinal Models

**Figure 5:**
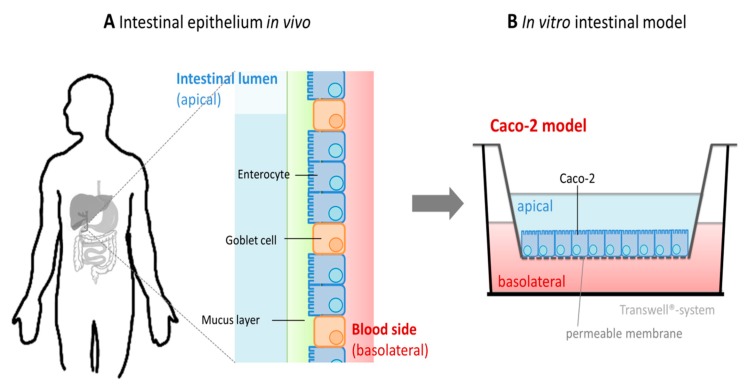
Schematic representation of the three-dimensional in vitro cellular intestinal model Caco-2. (**A**) The intestinal epithelium in vivo is mainly composed of enterocytes and goblet cells [255], which represents about 90% of intestinal cells of the brush border membrane [256,257]. These are covered by a viscoelastic gel: the mucus layer. This physical barrier is synthesized and secreted by goblet cells and serves as a protective layer for the underlying epithelium. (**B**) Three-dimensional Caco-2 monoculture in the “Transwell^®^ system”. The intestinal cell line Caco-2 is cultured in inserts on a permeable membrane, and, in most cases, composed of polycarbonate. This results in three compartments: an apical compartment representing the intestinal lumen, a basolateral side corresponding to the serosal surface of enterocytes, and the intestinal barrier formed by differentiated Caco-2 cells.

Until now, predominantly, the human Caco-2 cell model was used to elucidate intestinal zinc absorption and transport with in vitro studies. This model is widely employed to determine the absorption of various drug compounds as well as the uptake and transport kinetics of (micro-) nutrients [244,245,246,247] and is recognized by the FDA, giving promising correlations for fractional absorption of several drug components [248]. When cultured for 21 days, the epithelial colon carcinoma cell line Caco-2 differentiates into a state functionally and morphologically resembling human enterocytes [249,250]. They form an intact monolayer with important characteristics of the intestinal epithelium, including microvilli as well as tight junction proteins, and express several important proteins for intestinal transport [251,252]. In three-dimensional cultures, the cellular monolayer, seeded on Transwell inserts, forms an intact barrier mimicking the intestinal epithelium, whereas the apical transport chamber corresponds to the intestinal lumen and the basolateral side represents the serosal blood side [10] (Figure 5). By these means, a substance of interest, such as zinc, can be tracked from the apical compartment, its transport into the cells, and through the intestinal epithelium into the blood. Furthermore, this model can be combined with in vitro human digestion models to study bioavailability and absorption of the micronutrient from digested complex food samples [243,253,254].

While human zinc absorption and transport kinetics were characterized using three-dimensional Caco-2 models (Table 3), a two-dimensional culture of these cells was additionally applied to investigate zinc uptake parameters. Furthermore, this model was widely used to study the effects of various dietary food components on intestinal zinc bioavailability [96,243,247,258,259,260,261,262,263,264,265,266,267,268,269,270,271,272] and to elucidate the regulatory role of intestinal zinc transporters and metallothionein in zinc absorption [74,86,87,144,242,273,274,275,276,277,278,279,280]. Notably, the impact of dietary zinc on zinc transporters and metallothionein expression in Caco-2 cells is very well comparable to the homeostatic regulation of these proteins in human small intestine [86]. Supplementary Tables S3 and S4 summarize the studies’ design and outcome.

Aside of Caco-2-models, some studies on intestinal zinc uptake and transport were also done using the in vitro intestinal model IPEC-1 [281] and IPEC-J2 cells [242,275,282,283]. The non-transformed cell lines IPEC-1 and IPEC-J2 are derived from porcine intestine and are mainly used as in vitro models for pig intestine [284,285], but are described to resemble human enterocytes closer than any other animal-derived cell line [286].

### 5.2. Buffer Composition of In Vitro Cellular Intestinal Models

Speciation of zinc in cell culture medium or buffer severely affects its availability and cellular uptake in in vitro experiments [220,287]. A particular problem in this context is fetal calf serum (FCS), which proves to be an unpredictable factor due to its variability [288] and contains about 60% albumin in its protein fraction [289]. Notably, FCS is commonly used in cell culture [290] just as 10% FCS is used in many of the in vitro intestinal cell models presented in Table 3, which results in a final albumin concentration of 1.55 mg mL^−1^ [288] (corresponding to 24.2 µM) in the medium. Since albumin binds zinc with high affinity [221], apically added FCS or BSA severely impact its bioavailability for cells in vitro, as shown by decreased zinc toxicity [102] and uptake in the presence of these proteins [102,220,291,292].

Adding albumin to the apical side of in vitro intestinal models certainly does not represent the in vivo situation in the intestinal lumen and should be avoided when studying intestinal zinc absorption. When this protein is used as an apical component in some of the in vitro zinc transport studies based on three-dimensional Caco-2 models (Table 3), zinc transfer via the in vitro intestinal barrier is altered. Direct comparison of results from studies that were conducted in different laboratories is generally difficult. Nevertheless, comparing the outcome of zinc transport experiments using Caco-2 monocultures where zinc is applied on the apical side together with 10% FCS [67] with a study where no FCS is added to the apical side [103], the presence of 10% FCS diminishes cellular available zinc resulting in considerably smaller zinc transport rates (with 10% FCS: transport rate of apically applied 50 µM zinc after 4 h: 0.02 nmol cm^−2^ [67]; without FCS: transport rate of 50 µM zinc after 4 h: 0.95 nmol cm^−2^ [102]). In fact, the authors of this study [67] applied 10% FCS on the apical side of their intestinal model to mimic the luminal protein matrix during transport studies. However, the presence of intact proteins does not accurately reflect the luminal environment in vivo, as they would have been digested into smaller molecules (peptides or amino acids). Apical addition of zinc together with in vitro digested albumin significantly increases the zinc bioavailability for Caco-2 cells compared to undigested protein [102].

In contrast to the apical side of in vitro models, where albumin should be excluded, serum albumin is the main zinc transporting protein in plasma. As already discussed, serum albumin is an important physiological factor for intestinal zinc absorption and influences zinc excretion from enterocytes into the blood circulation [102]. The zinc-accepting role of albumin during the absorption process emphasizes the relevance of this basolateral constituent, which, consequently, has to be added to the basolateral compartment of the intestinal models to resemble the blood in vivo.

Some of the in vitro zinc transport studies performed with Caco-2 models (Table 3) use cell culture medium with 10% FCS for the basolateral compartment [67,68,71,102,103,239]. This FCS concentration, however, yields only 3–5% of the serum albumin concentration in vivo. Although some in vitro studies apply zinc to the basolateral compartment, mainly to investigate the serosal zinc uptake into the intestinal epithelium [66,67,68,239], the apical zinc transport in the presence of physiological zinc and albumin concentrations on the basolateral side has not been investigated in these studies. In this case, the zinc content on the basolateral side originated predominantly from FCS [67,68,71,239]. The exact basolateral zinc concentrations in these studies are unknown. FCS generally contains higher amounts of zinc than cell culture medium [293], but its total zinc content varies significantly, which leads to final zinc concentrations between 3 [74] and 14 µM [294] in complete media. The addition of albumin, apart from the amount already present in FCS, to the basolateral side of the in vitro model, however, was only performed in four of the in vitro transport studies with Caco-2 monocultures or Caco-2/HT-29-MTX co-cultures, respectively, by applying very low (2.5 mg mL^−1^) [238] or physiological albumin concentrations (5% BSA, corresponding to 50 mg mL^−1^ albumin [71], and 30 mg mL^−1^ albumin [102,103]).

### 5.3. Cellular Composition of In Vitro Cellular Intestinal Models

The in vitro Caco-2 model lacks one very important factor of the intestinal epithelium, which is the mucus layer. The intestinal epithelium in vivo is not only composed of enterocytes, but also includes goblet cells, producing and secreting mucins, covering the whole gastrointestinal tract (in detail, reviewed in References [214,295]). As already discussed, the mucus layer provides an important physiological luminal factor for intestinal zinc uptake and absorption [103,212,213]. Consequently, the application of a mucus layer or mucin-producing cells in in vitro models to study intestinal zinc absorption should not be neglected. Simulation of the mucus layer by adding isolated (porcine) mucins on top of three-dimensional Caco-2 monocultures was previously critically discussed in connection with iron transport and zinc uptake studies [103,254,296]. These mucins do not display similar viscoelastic and gel forming properties of the gastrointestinal mucus layer in vivo because of their isolation and purification process [297,298]. Moreover, isolated mucins do not simulate transmembrane mucins, which represent an important fraction of the mucus layer in vivo [295].

Co-culturing Caco-2 cells together with the goblet cell line HT-29-MTX yields an in vitro model that not only constitutes the two main cell types of the intestinal epithelium [255], but also contains mucus covering the whole cell layer [254]. Moreover, this co-culture does not only improve the in vitro intestinal Caco-2 model regarding the presence of a mucus layer, but was also reported to optimize the cellular permeability of conventional Caco-2 monocultures [299,300] and is considered a more physiological in vitro model [256,301]. The Caco-2/HT-29-MTX model is well characterized [299,302,303,304] and was already used to investigate the absorption of different metal species [254,305,306,307], the effect of nanoparticles on nutrient absorption [308], and bacterial adhesion [309]. Recently, a Caco-2/HT-29-MTX model, optimized with respect to its buffer composition and basolateral serum concentration, was applied to study the absorption of zinc via the human intestinal epithelium in the presence of a mucus layer [102,103]. This in vitro intestinal model showed enhanced net absorption and transport rates of apically applied physiological zinc concentrations (25–100 µM) compared to conventional Caco-2 monocultures [67,120,243] and comparable amounts of actually transported zinc to those estimated in vivo [105].

Regarding the cellular composition of the intestinal epithelium and its vicinity, it would certainly be worthwhile to analyze zinc transport via the intestinal barrier in the presence of other intestinal cells in addition to enterocytes and goblet cells. There are various three-dimensional Caco-2 co-cultures or even triple-co-cultures of Caco-/HT-29-MTX with different cell lines, including immune cells such as THP-1 human macrophages or the M-cell-resembling Raji B cell line (reviewed in Reference [310]). Additionally, using a triple-co-culture of Caco-2/HT-29-MTX with peripheral blood mononuclear cells (PBMC) [311,312] would provide the option to study the impact of leukocytes in the blood serum on intestinal zinc absorption.

### 5.4. Comparison of In Vitro Cellular Intestinal Models with the In Vivo Situation

Applying in vitro models to mimic processes in vivo requires critical consideration of their limitations. Even though current research aims to use improved and more physiological in vitro intestinal models, like the mucin-producing in vitro co-culture Caco-2/HT-29-MTX [105,256], for studying zinc transport, there are differences in the in vivo intestinal epithelium that have to be considered. Two important physical factors in the intestine in vivo are lacking in the in vitro models: intestinal and blood fluid flow as well as peristaltic motions. Intestinal peristalsis enables movement of chyme along the intestine and increases mechanical degradation of food components [255], which is important for the digestion process and availability of nutrients for absorption. On the other hand, absorbed zinc is bound to albumin in vivo and continuously transported within the blood circulation, which distributes the cation throughout the whole body [23]. This sink is missing in vitro and absorbed zinc accumulates in the basolateral compartment of three-dimensional cell models.

Table 3 summarizes studies of zinc absorption using three-dimensional Caco-2 mono- and co-cultures, which depicts parameters of cell models including buffer composition and the main outcome of the study. Regardless of the detailed experimental setting, almost all transport studies obtained with Caco-2 models observe saturable apical zinc uptake and transport kinetics. Two different studies using Caco-2 monocultures observed non-saturable zinc uptake from the apical side, both using regular cell culture medium with 10% FCS for their apical zinc treatment [67,68] and either of them unphysiologically high zinc concentrations [68]. Additionally, the transported amount of the micronutrient to the basolateral side is not comparable to that in vivo [67]. This underlines the importance of applying zinc corresponding to physiological concentrations in the intestinal lumen in vivo, particularly when analyzing transport and uptake kinetics, in order to prevent artefacts.

K_m_ values for zinc uptake of 41 µM [66] or 11.7 µM [71] obtained with Caco-2 are in the same order of magnitude as those determined with in vitro rat intestines (K_m_ = 10–12 µM [71]), rat perfused intestines (K_m_ = 32 µM [38]; K_m_ = 29 µM [73], K_m_ = 55 µM [70]), or brush-border membrane vesicles from pig (K_m_ = 67 µM [196] or rat (K_m_ = 24 µM [313]). Accordingly, Caco-2 cells seem well suited for studying intestinal zinc uptake.

Compared to the fractional zinc absorption of in vitro cellular models (~2–6%) [102,103,243], the estimated net absorption of 16–50% for humans in vivo [7,30,33,75,76,77] is significantly higher. Notably, most of these in vivo studies investigate fractional zinc absorption from meals containing dietary ligands that affect zinc bioavailability in the intestinal lumen [36], whereas, in the in vitro studies, zinc is mainly added as liquid solutions and without a food matrix. In vivo studies estimating the fractional zinc absorption from liquid solutions with comparable zinc concentrations to those applied in the in vitro studies are scarce [57,58]. Even though the application of improved and more physiological in vitro intestinal models, which include a mucus layer and basolateral added serum albumin, result in higher zinc net absorption [102], the amounts represent only about 10% of the fractional zinc absorption in vivo when analyzing the absorption of zinc levels typically found in the intestinal lumen after a meal [7,105].

To explain this discrepancy, the ratio of intestinal liquid per absorption area has to be taken into account. The absorption area of in vitro three-dimensional intestinal models (commonly using Transwell inserts with an area of 1.12–4.67 cm^2^) is a lot smaller than the intestinal epithelium (about 30,000 cm^2^ [314]). This rough assessment does, however, not include the actual amount of absorptive enterocytes per absorption area and disregards the increase in absorption area in vitro by microvilli formation of Caco-2 cells. The volume of intestinal liquid in lumen in vivo amounts to around 3 L [255] (corresponding to 0.1 mL cm^−2^), whereas, in an in vitro model with an absorption area of 1.12 cm^2^ (typical apically added volume: 500 µL), the volume to area ratio is 0.45 mL per cm^2^, which leads to a 4.5-fold higher apically applied liquid volume per cm^2^ absorption area in vitro. Hence, the total amount of zinc available for transport per absorption area in vitro is greater than in vivo, which distorts fractional absorption. Assuming that expression and activity of the main zinc transporters in Caco-2 cells in vitro correspond to those in vivo, which we certainly do not know yet, a higher ratio of zinc per cm^2^ absorption area in the in vitro model could explain a smaller fractional absorption. Regrettably, adjusting the liquid volume applied into apical chambers of in vitro intestinal models to the volume per cm^2^ ratio in vivo (0.1 mL cm^−2^) is not an option, as it would impair cellular viability.

Remarkably, the amounts of zinc that are actually transported per cm^2^ absorption area to the basolateral side of three-dimensional in vitro models are comparable to quantities absorbed per cm^2^ of intestinal epithelium into the blood circulation in vivo. Table 4 depicts estimated amounts of actual transported zinc in vitro and in vivo, using data of an in vitro Caco-2/HT-29-MTX co-culture [102] and a human in vivo study by Hunt et al. [7], where comparable zinc concentrations are applied with a meal. This also applies to some of the studies using in vitro Caco-2 mono-cultures [66,103,120] and Caco-2/HT-29-MTX co-cultures [103] presented in Table 3. Consequentially, when comparing the results from in vitro cellular intestinal models with the in vivo zinc absorption, it is more relevant to correlate the actual amount transported per cm^2^ absorption area than net absorption of zinc.

## 6. Analytical Approaches to Studying In Vitro Zinc Absorption and Bioavailability

Zinc absorption and bioavailability in humans is mostly analyzed with (stable) isotope tracer techniques, primarily measuring fractional zinc absorption [315]. In earlier studies, the zinc radioisotope ^65^Zn was also used to investigate zinc homeostasis [316] and bioavailability in humans [317], but is currently replaced by non-radioactive and stable isotopes [318] and solely employed in vitro [66,71,79].

**Figure 6:**
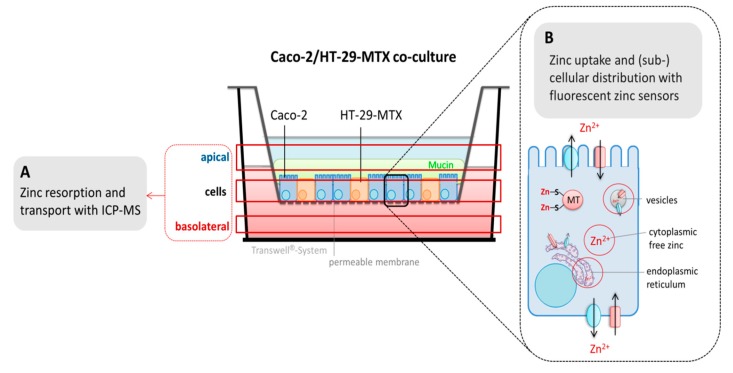
Application of in vitro cellular intestinal models to study intestinal zinc transport. Schematic representation of the three-dimensional intestinal Caco-2/HT-29-MTX co-culture model. (**A**) Zinc is quantified in all three compartments (apical, cellular, basolateral) with conventional analytical approaches, such as inductively coupled mass spectrometry (ICP-MS) or flame atomic absorption spectrometry (FAAS). (**B**) The application of chemical-based or protein-based fluorescent zinc sensors in enterocytes provides additional information about the subcellular distribution of the micronutrient upon its uptake into the cell. These sensors bind intracellular free zinc and track small changes of this zinc moiety. Depending on the subcellular localization of the sensor, the cytoplasmic free zinc pool or free zinc in organelles, such as vesicles and the endoplasmic reticulum (circled in red) can be investigated.

In three-dimensional in vitro cellular intestinal models, the quantity of the metal in the apical and basolateral compartment as well as the cellular zinc content is analyzed to determine the amount of absorbed and actually transported zinc to the blood side (Figure 6A). Thus, transport kinetics, net absorption, and bioavailability of luminally-added zinc species are investigated. Aside of (stable) isotope techniques, zinc is generally quantified with inductively coupled mass spectrometry (ICP-MS), inductively coupled plasma optical emission spectrometry (ICP-OES), or atomic absorption spectrometry (AAS) [319]. In addition to measuring the most abundant stable zinc isotopes, ^64^Zn or ^66^Zn, with ICP-MS, ^70^Zn was recently used to determine cellular zinc uptake kinetics while simultaneously distinguishing between cellular basal zinc levels and zinc that was actually absorbed by the cells [320]. Hence, applying this method in in vitro intestinal models would provide a fruitful approach for scrutinizing enterocyte homeostasis of this micronutrient during zinc absorption.

Aside of determining enterocyte zinc uptake or transport, in vitro intestinal models offer the great opportunity to scrutinize subcellular compartmentalization of the metal by providing additional information about its disposition and cellular availability after its absorption into enterocytes (Figure 6B). Fluorescent zinc sensors are a versatile tool for analyzing small subcellular changes of free zinc [321]. These sensors bind free or mobile zinc, which represents a particularly small fraction of the cellular zinc content.

Fluorescent zinc sensors can be classified into low molecular weight (LMW) sensors (or chemical sensors) and genetically-encoded biosensors [321]. Below, their function and application in intestinal cell models in vitro as well as advantages and disadvantages of the two classes of sensors are briefly summarized.

The principle of most LMW sensors is based on photo-induced electron transfer (PET) between the fluorophore and a chelating unit, which, in case of a non-radiometric sensor, quenches fluorescence when no metal is present. Metal binding leads to disruption of PET and increase of fluorescence (reviewed in detail in Reference [322]). After entering the cells by passive diffusion, changes in fluorescence upon binding of intracellular free zinc can be analyzed with fluorescence spectrometric methods to quantify free zinc or use fluorescence microscopy to image spatial distribution of the cation [322,323].

The basic concept of genetically-encoded sensors is comparable to LMW probes, which results in measurable fluorescence changes upon zinc binding. Various ratiometric biosensors have been developed based on Förster resonance energy transfer (FRET) between two fluorescent molecules [324,325,326,327]. Generally, these fusion proteins are composed of two fluorescent domains and a metal binding site connected by a flexible linker. Emission wavelength of the donor fluorescent domain overlaps with the excitation wavelength of an acceptor domain, which results in a FRET signal when these fluorescent molecules are in spatial proximity. Conformational changes upon zinc binding consequentially lead to a shift of FRET signals [322]. Moreover, Aper et al. created the first set of zinc-dependent bioluminescence resonance energy transfer (BRET) biosensors, where, instead of a donor fluorophore, a stable NanoLuc luciferase domain is exciting the acceptor fluorescent molecule via BRET [328]. Most recently, Palmer et al. developed a biosensor based on a single fluorescent protein [329]. In contrast to low molecular weight sensors, these probes are genetically encoded and, thus, transfected as plasmids into the cells [325]. Consequently, the cell produces the sensor controlling its subcellular concentrations and distribution, which makes them particularly convenient for long-term measurements and less invasive than chemical probes [97,321]. Introduction of BRET-based biosensors circumvents some disadvantages of FRET-sensors by including autofluorescence and photobleaching of fluorophores due to the illumination of the sample, which is necessary for the excitation of the donor domain [328]. Furthermore, FRET analysis requires an elaborate technical approach based on laser scanning microscopy determining FRET or fluorescence life time imaging (FLIM)-FRET, and is almost exclusively limited to analyzing single cells [330]. BRET-based biosensors can be employed in high throughput screening assays using bioluminescence plate readers [328,331,332].

Of particular interest are zinc biosensors with organelle-specific targeting, which accumulate in distinct organelles within the cell, such as mitochondria, Golgi apparatus, endoplasmic reticulum, and cell membranes [324,333,334,335]. Although subcellular distribution of LMW sensors is generally not easy to control, chemical probes with specific cellular targeting have already been successfully developed [336,337,338].

In terms of application of these sensors in human intestinal cell lines to either measure zinc uptake or analyze its subcellular distribution, low molecular weight sensors Zinpyr-1 [96,102,103], Fluozin-3 [156,339,340], and Zinquin [339] were already used in Caco-2 and the colorectal adenocarcinoma cell line HT-29 (Table 5). Recently, a Caco-2 clone stably expressing the FRET biosensor eCalwy-5, originally generated by Merkx and co-workers [325], was established [101] by providing a well characterized in vitro intestinal model to study intestinal zinc uptake. It can be co-cultured with other cell lines, such as HT-29-MTX, allowing it to specifically analyze free zinc in Caco-2 cells of co-cultures. Consequentially, the micronutrient can be tracked after its absorption into enterocytes in the presence of goblet cells and a mucus layer, while LMW probes would always stain the entire model.

## 7. Conclusions and Outlook

Regarding human intestinal zinc absorption, there are several points that still await to be answered. Accordingly, zinc homeostasis of enterocytes and the molecular processes inside these cells during intestinal zinc absorption have to be further investigated. In particular, the zinc transfer through enterocytes upon its absorption, its subsequent basolateral release into the blood circulation, and the involvement of a zinc-binding or -trafficking protein in this process, other than MTs, need to be studied in more detail. In addition, the involvement of zinc transporters in cytoplasmic organelles of enterocytes (like ZnT-2, ZnT-4, ZnT-6, and ZnT-7) in cellular zinc trafficking and homeostasis has to be addressed. In recent years, the relevance of post-transcriptional modifications of intestinal zinc transporters has been recognized to play an important role in regulating their function as well as transport activity and needs further clarification. Moreover, the role of systemic and humoral factors in regulating enterocyte zinc uptake from the intestinal lumen and excretion via the basolateral exporter ZnT-1 into the blood has to be comprehensively elucidated.

To tackle these challenges, the application of in vitro cellular intestinal models has exceptional potential. As outlined in detail in this review, these models provide a standardized platform to not only analyze zinc absorption and transport kinetics and bioavailability from in vitro digested food samples, but also to elucidate the regulatory parameters of human zinc absorption and transport on a molecular level, offering several advantages compared to other intestinal models. Prospective implementation of these in vitro intestinal microenvironments could include analysis of diet-derived factors impacting intestinal zinc uptake as well as the fast and cost-efficient screening of zinc bioavailability from novel food products and zinc complexes. Consequentially, the role of amino acids and peptides in luminal availability and subsequent absorption of the metal can be studied. Knowledge about the bioavailability of zinc from complex food matrices could be included in nutrition surveys. Most of these are currently only considering the total zinc content of the respective diet.

As discussed in detail in this review, the luminal and basolateral constituents as well as cellular composition of in vitro cellular intestinal models are crucial for investigating zinc absorption and must represent the in vivo human intestinal epithelium and its vicinity as close as possible. Still, there remain important differences to the intestinal epithelium in vivo, as not all cell types present in the epithelium are incorporated in the models, and the intestinal and blood fluid flow and peristaltic motions are not considered. Importantly, it is more relevant to correlate the actual amount of transported metal per absorption area than the net absorption in vitro and in vivo because there are considerable differences of the ratios of liquid volume per absorption area. Hence, several factors and limitations have to be considered when using in vitro intestinal models to study the intestinal zinc absorption, and should be addressed during further development of improved in vitro models.

Taken together, investigating these future perspectives with in vitro cellular intestinal models would not only enhance the current knowledge on intestinal zinc bioavailability and absorption as well as on molecular regulatory parameters of its luminal uptake and transport into the blood circulation, but also contribute to the overall understanding of enterocyte zinc homeostasis in addition to the hitherto obtained results.

## Figures and Tables

**Table 1:** Zinc and phytate content, as well as phytate: zinc-molar ratios of selected plant-based foods.

Food Group	Food	Zinc Content (mg/100g)	Phytate Content (mg/100g)	Phytate: Zinc Molar Ratio	Reference
Seeds and nuts	Sesame seeds	2.48	1525	60.9	[176]
Beans and lentils	Lentils	3.03–4.02	747–961	18.5–27.8	[177]
Whole grain cereals	Durum wheat	2.4–4.8	460–952	16.9–23.6	[178]
Vegetables	Sweet potato (boiled)	0.30	31–37	12.3–15.2	[179]
Fruit	Passion fruit	0.41–0.48	77.2–86.8	15.3–20.6	[179]
Refined cereals	Refined wheat flour	0.52	37	6.47	[180]

Phytate: Zinc-Molar ratio was estimated based on (mg phytate/660)/(mg zinc/65.4).

**Table 2:** Comparison of intestinal models to study intestinal zinc transport and absorption.

Intestinal Model	Method	Main Outcome	Advantage	Disadvantage	References
Ussing chamber	- ex vivo: Isolated epithelium from pig, rat intestinal tract mounted into the Ussing chamber	- zinc transport rates from mucosa to serosa - effect of zinc on epithelial secretion and electrophysiological response	- zinc transport via ex vivo intestinal epithelium, including different intestinal cell types and mucus layer, can be investigated	- not easy to standardize because of inter-individual differences [237]	[225,226,227,228,229,230,231]
Everted gut sac	- ex vivo: isolated rat intestinal segments (duodenum, jejunum, colon)	- zinc uptake by the intestinal segment	- absorptive properties of the distinct intestinal segments	- not easy to standardize because of inter-individual differences - peristaltic and fluid flow is missing	[60,61]
Perfused intestine	- ex vivo or in situ: isolated segments from intestine, vascularly and/or luminally perfused	- fractional zinc absorption - zinc transport kinetics	- zinc transport kinetics via the intestinal epithelium in a physiological vicinity (mucus layer, various intestinal cells)	- expensive, elaborate	[38,70,115,234]
Brush border membrane vesicles	- in vitro: BBM vesicles are prepared from isolated intestinal mucosa from rat or pig	- zinc transport kinetics - interactions of zinc with BBM	- suitable system to characterize and estimate transport kinetics that occur solely via BBM	- mucus layer is removed during preparation of BBM - part of intracellular zinc homeostasis in regulating transport via BBM is disregarded	[72,232,233]
In vitro intestinal cell model	- In vitro intestinal cells cultivated in three-dimensional transwell dishes - Caco-2 mono-cultures - Caco-2/HT-29-MTX co-cultures - hiPSC - IPEC-1, IPEC-J2 (porcine intestinal model)	- fractional zinc absorption and zinc transport kinetics - molecular parameters of zinc uptake and absorption	- standardized microenvironment to study molecular parameters as well as absorption kinetics - combinable with in vitro digestion models to study zinc bioavailability from digested food samples	- mostly using in vitro cell lines, that are tumorigenic and transformed - basolateral sink and fluid flow is missing - apical and basolateral peristaltic is missing	[66,67,68,71,96,102,103,120,156,238,239,240,241,242,243]

BBM, brush border membrane; hiPSC, human induced pluripotent stem cells.

**Table 3:** Zinc transport studies using in vitro intestinal models.

Cell Model	Incubation Parameter	Type of Zinc	Main Outcome	Reference
Caco-2 cells Cultivation time: 14 d 3D Transwell (PC membrane) 14 d	ZnCl_2_ 20 µM (Kinetic 0–50 min) 0–100 µM (10 min) (in salt buffer on apical and basolateral side) Inhibitor: ouabain, vanadate, dinitrophenol, sodium cyanide, ammonium vanadate Potential zinc ligands: histidine, cysteine, proline, glutathione	radioactive zinc (^65^Zn)	- cellular zinc uptake is saturable process - K_m_ = 41 µM V_max_ = 0.3 nmol/cm^2^/10 min - basolateral zinc uptake was partially inhibited (30%) by ouabain and vanadate, which suggests an involvement of the (Na-K)-ATPase in serosal uptake - apical zinc uptake was not affected by metabolic inhibitors and ligands - basolateral zinc uptake (50 min) ~ 0.47 nmol/cm^2^ - zinc transport ~ 0.8 nmol/cm^2^ (20 µM, after 50 min) - transport from basolateral to apical is higher than from the apical to the basolateral compartment	[66]
Caco-2 cells Cultivation time: 18–21 d 3D Transwell	ZnSO_4_ 10–1000 µM (for 90 min) 10 nM 1α,25-dihydroxyvitamin D_3_ (preincubation for 72 h) + 100 µM ZnSO_4_ (for 90 min) Apical: MES-buffer with NaCl, KCl, MgSO_4_, CaCl_2_, glutamine, glucose, Basolateral: 2.5 mg/mL BSA in Hepes with NaCl, KCl, MgSO_4_, CaCl_2_, glutamine, glucose,	radioactive zinc (^65^Zn)	- saturable zinc uptake kinetic up to 1000 µM - K_m_ = 226 µM - zinc transport rate (after 90 min): ~10 µM: ~0.12 nmol/cm^2^ ~50 µM: ~0.25 nmol/cm^2^ - zinc transport increased in vitamin D_3_ incubated cells - *mt-2a* mRNA and protein was increased with greater zinc concentrations - *Crip* mRNA (30% less expressed in Caco-2 cells than in rat mucosa) was decreased by vitamin D_3_ treatment	[238]
Caco-2 cells Cultivation time: 21 d 2D, 3D Transwell (PE membrane)	zinc species: n.a. 1–200 µM (in DMEM + 10% FCS on apical and basolateral side) for 0–30 h	radioactive zinc (^65^Zn)	- saturable zinc uptake at the basolateral membrane - apical zinc uptake and zinc transport, both from apical to basolateral and vice versa, were not saturable - higher transport from apical to basolateral - transport rate 50 µM: 6 pmol/h/cm^2^ - transport from apical to basolateral was independent from basolateral zinc concentration - study indicates that zinc uptake and transcellular movement are different at the apical and basolateral side	[67]
Caco-2 cells Cultivation time: 14–16 days of 3D Transwell (Polyethylene terephthalate membrane)	ZnSO_4_ 0–1000 µM (in DMEM + 10% FCS on apical) and 0–450 µM (in DMEM + 10% FCS on basolateral side) for 24 h	total Zn	- applied 0–1000 µM zinc on apical or 7–450 µM zinc basolateral side - transport occurs from both sides to the other compartment - accumulation in the cells was low, particularly when zinc was added on the apical side - zinc toxicity on cell viability and integrity of the intestinal barrier (TEER) 0–2000 µM zinc: - observed higher toxicity when adding high zinc concentrations to the basolateral side	[68]
Caco-2 cells Cultivation time: 18–21 days of 3D Transwell (PC)	ZnCl_2_ 50–200 µM (in serum free medium on apical and basolateral side) for 6 h, 12 h, and 24 h	radioactive zinc (^65^Zn)	- zinc transport an MT secretion (HPLC analysis) - this study suggest that MT is secreted into the gastrointestinal lumen and plays a role in intestinal zinc uptake - zinc transport (after 6 h) - 100 µM: ~2.0 nmol/cm^2^	[120]
Caco-2 cells Cultivation time: 21 d 3D (PES-HD membranes)	ZnSO_4_ 5 µM or 25 µM (in DMEM + 10% FCS on apical and basolateral) (preincubation for 7 d)	radioactive zinc (^65^Zn)	- zinc uptake and transport were measured in both apical (AP) and basolateral (BL) directions - rate of apical zinc uptake and transport rate to basolateral was lower in cells pretreated 25 µM zinc - basolateral zinc release was higher in cells treated with 25 µM - cellular zinc uptake 2–3 nmol mg^−1^ protein - induction of MT (analyzed using radiolabeled cadmium) was zinc-dependent, increasing with zinc concentration	[239]
Caco-2 cells Cultivation time: 21 d 3D Transwell (PC)	ZnSO_4_ 15.6–500 µM (apical: KHB buffer, basolateral: KHB-buffer + 5% BSA)	total Zn	- comparison with zinc transport across isolated rat small intestine - rat: K_m_ = 10–12.1 µM - Caco-2 K_m_ = 11.7 µM V_max_ = 31.8 pmol min^−1^ cm^−2^ - transport across Caco-2 monolayers is carrier-mediated and energy-dependent - zinc transport into basolateral chamber followed a saturated process - transport rate: 50 µM: 39 pmol min^−1^ cm^−2^ - mRNA expression of *zip-4*, *zip-5*, *znt-1*, *mt1*, *mt2* in duodenum, jejunum, and ileum of isolated rat small intestine	[71]
Caco-2 cells Cultivation time: 17 days 3D Transwell (Polytetrafluoroethylene)	ZnSO_4_ 100 µM (serum free medium on apical and basolateral side) for 3–24 h 1 µM hepcidin	stable zinc isotope (^67^Zn)	- hepcidin reduces basolateral zinc export by post-translationally downregulation of ZnT-1 - cells incubated with hepcidin showed less zinc export while cellular zinc and *mt*-*1a* mRNA increased, cell surface ZnT-1 as well as ZnT-1 protein decreased - hepcidin might play a role in controlling zinc absorption and enterocyte subcellular zinc pools	[156]
Caco-2/HT-29-MTX co-culture Cultivation time: 21 days 3D Transwell (PC)	ZnSO_4_ 0–100 µM (apical: serum-free transport buffer, basolateral: DMEM +10% FCS + 0 or 30 mg mL^−1^ BSA) for 8 h	total Zn	- albumin has a role in in vitro zinc absorption as a basolateral zinc acceptor - cellular uptake is not significantly different with or w/o basolateral added albumin - basolateral serum albumin enhances cellular zinc export to the basolateral side - fractional absorption (25–100 µM): w/o BSA: ~2% with BSA: 5.8–2.9% - zinc transport rates (0–100 µM): w/o BSA: 0.1–2.2 nmol cm^−2^ with BSA: 1.1–3.6 nmol cm^−2^	[102]
Caco-2/HT-29-MTX co-culture and Caco-2 monoculture Cultivation time: 21 days 3D Transwell (PC)	ZnSO_4_ 0–100 µM (apical: serum-free transport buffer, basolateral: DMEM + 10% FCS + 30 mg mL^−1^ BSA) for 4 h	total Zn	- intestinal mucins influence cellular zinc uptake and zinc transport - results suggest that mucins facilitate zinc uptake into enterocytes and act as a zinc delivery system - mucins are an integral part of intestinal zinc absorption - fractional absorption (25–100 µM): monoculture: 1.6–0.9% co-culture: 4.2–1.9% - zinc transport rates (0–100 µM): monoculture: 0.3–1.3 nmol cm^−2^ co-culture: 1.1–2.3 nmol cm^−2^	[103]

3D, three-dimensional. BSA, bovine serum albumin. DMEM, Dulbecco’s Modified Eagles Medium. FCS, fetal calf serum. HBSS, Hank’s Balanced Salt Solution. HD, high density. KHB, Krebs-Henseleit buffer. n.a., not available. PC, polycarbonate. PE, polyethylene. PES, polyester. Zn, zinc.

**Table 4:** Total amounts of absorbed zinc in vivo and in the in vitro intestinal model Caco-2/HT-29-MTX.

**In Vitro Caco-2/HT-29-MTX [102] (Absorption Area = 1.12 cm^2^, Volume: 500 µL)**			
**Apical Zinc**	**Fractional Absorption (%)**	**Absorbed Zinc (µg/Total Absorption Area)**	**Absorbed Zinc (µg cm^−2^)**
100 µM = 3.23 µg/1.12 cm^2^	2.9	0.09	0.08
25 µM = 0.82 µg/1.12 cm^2^	5.8	0.05	0.04
**In vivo [7] (Absorption Area = ~** **30 m^2^ [314], Volume: ~3 L [255])**			
**Apical Zinc**	**Fractional Absorption (%)**	**Absorbed Zinc (µg/Total Absorption Area)**	**Absorbed Zinc (µg cm^−2^)**
17 mg/30 m^2^ = 86 µM	24	4080	0.14
4.3 mg/30 m^2^ = 21 µM	49	2100	0.07

**Table 5:** Application of chemical-based and protein-based fluorescent sensors to study free zinc in enterocytes.

Cell Model	Sensor	Incubation Parameter	Main Outcome	Reference
HT-29 Cultivation time: - proliferating cells: 24 h - resting cells: 48 h (serum depleted) - differentiated cells: 6 days (first 72 h with sodium butyrate); 2D	FluoZin-3 (K_d_ = 8.9 nM) Newport Green (K_d_ = 30 µM) (low molecular weight sensors)	- sensor pre-incubation: 0.3–5 µM FluoZin-3 or 5µM Newport Green for 30 min in DPBS - spectrofluorometer	- free zinc in HT-29 0.6–1.2 nM for proliferating, resting or differentiated cells - a surplus of zinc-binding proteins buffer the intracellular free zinc concentration and guarantee a stable zinc homeostasis	[340]
Caco-2/TC7 Cultivation time: 15-17 days; 2D	FluoZin-3 (K_d_ = 15 nM) Zinquin (low molecular weight sensors)	- sensor pre-incubation: 1 µM FluoZin-3; 25 µM Zinquin - samples were fixed with paraformaldehyde prior staining - fluorescence microscope	- both sensors accumulate in vesicle-like structures - imaging of free zinc distribution and tight junction formation in enterocytes	[339]
HT-29 Cultivation time: n.a.; 2D	Newport Green (low molecular weight sensor)	- sensor pre-incubation: 5 µM Newport Green for 30 min in assay buffer ^a^ - fluorescence microplate reader	- increase of intracellular free zinc levels after zinc treatment are lower than changes in total cellular	[341]
Caco-2 Cultivation time: 17 days; 2D	FluoZin-3 (K_d_ = 15 nM) (LMW sensor)	- sensor pre-incubation: 1 µM FluoZin-3 for 1 h in OptiMEM, - fluorescence microscope and microplate reader	- sensor accumulates in vesicles - basal free zinc decreases after treatment with hepcidin	[156]
Caco-2 Cultivation time: 10 days; 2D	Zinypr-1 (K_d_ = 0.7 nM) (low molecular weight sensor)	- sensor pre-incubation: 50 µM Zinpyr-1 for 1 h in PBS - fluorescence microscope	- zinc uptake from different zinc-complexes with amino acids	[96]
Caco-2-eCalwy Cultivation time: resting state; 2D	eCalwy-5 (K_d_ = 1.85 nM) (Genetically encoded protein-based sensor)	FRET and FLIM-FRET measurements using low molecular weight (LSM) in assay buffer ^b^	- eCalwy protein is mainly localized in the cytoplasm of the Caco-2-eCalwy clone - cytoplasmic free zinc was estimated to be around ~2 nM	[101]
Caco-2 Cultivation time: 21 days; 2D	Zinpyr-1 (K_d_ = 0.7 nM) (low molecular weight sensor)	- sensor pre-incubation: 2.5 µM Zinpyr-1 for 30 min in assay buffer + 0.3% BSA - fluorescence microplate reader	- sensor accumulates in cellular vesicles - basal free zinc was estimated to be ~0.2 nM	[102]
Caco-2 Cultivation time: 21 dHT-29, HT-29-MTX Cultivation time:7 days; 2D	Zinypr-1 (K_d_ = 0.7 nM) (LMW sensor)	- sensor pre-incubation: 2.5 µM Zinpyr-1 for 30 min in assay buffer + 30% BSA - fluorescence microplate reader	- sensor accumulates in cellular vesicles (HT-29, HT-29-MTX, Caco-2) - basal free zinc was estimated to be ~0.5 nM in HT-29-MTX, 0.8 nM for HT-29	[103]

2D, two-dimensional. BSA, bovine serum albumin. DMEM, Dulbecco’s Modified Eagles Medium. DPBS, Dulbecco’s phosphate buffered saline. FCS, fetal calf serum. FLIM, fluorescence lifetime imaging microscopy. FRET, Förster resonance energy transfer. HBSS, Hank’s Balanced Salt Solution. HEPES, 4-(2-hydroxyethyl)-1-piperazineethanesulfonic acid. LSM, laser scanning microscope. n.a., not available. PBS, phosphate buffered saline. ^a^ 120 mM NaCl, 5.4 mM KCl, 0.8 mM MgCl_2_, 20 mM Hepes, 15 mM glucose, 1.8 mM CaCl_2_, 10 mM NaOH, pH 7.4. ^b^ 120 mM NaCl, 5.4 mM KCl, 5 mM glucose, 1 mM CaCl_2_, 1 mM MgCl_2_, 1 mM NaH_2_PO_4_, 10 mM HEPES, pH 7.35.

## Supplementary Materials

The following are available online at https://www.mdpi.com/2072-6643/12/3/762/s1. Table S1: Zinc content of the human body. Table S2. Recommended daily allowance for dietary zinc intake for selected life-stages. Table S3. Application of human in vitro intestinal models to study zinc-dependent gene expression in enterocytes. Table S4. Application of in vitro Caco-2 monocultures to investigate the effect of dietary factors on zinc bioavailability.

## Abbreviations

2D	two-dimensional
3D	three-dimensional
λ_em_	emission wavelength
λ_ex_	excitation wavelength
BRET	bioluminescence resonance energy transfer
BSA	bovine serum albumin
DGE	German Society for Nutrition, ger. *Deutsche Gesellschaft für Ernährung*
DMEM	Dulbecco’s Modified Eagles Medium
DMT-1	divalent metal transporter
EDTA	ethylene-diamine-tetra-acetic acid
EFSA	European Food Safety Authority
EHS	Engelbreth-Holm-Swarm cells
FAAS	flame atomic absorption spectrometry
FCS	fetal calf serum
FLIM	fluorescence lifetime imaging microscopy
FRET	Förster resonance energy transfer
HD	high density
HBSS	Hanks’ Balanced Salt Solution
HEPES	4-(2-hydroxyethyl)-1-piperazineethanesulfonic acid
HSA	human serum albumin
ICP-MS	inductively-coupled plasma mass spectrometry
ICP-OES	inductively-coupled plasma optical emission spectrometry
IP	inositolphosphate
K_m_	half saturation constant
KHB	Krebs-Henseleit buffer
LIM	Lin-11, Isl-1, Mec-3
LMW	low molecular weight
LSM	laser scanning microscope
mRNA	messenger ribonucleic acid
MEM	minimum essential medium
MT	metallothionein
MTF-1	metal regulatory transcription factor 1
n.a.	not available
PBMC	peripheral blood mononuclear cells
PBS	phosphate buffered saline
PC	polycarbonate
PE	polyethylene
PES	polyester
PET	photo-induced electron transfer
*q*PCR	quantitative real time polymerase chain reaction (PCR)
RING	really interesting new gene
SLC	solute carrier
TEER	transepithelial electrical resistance
TJ	tight junction
TPEN	*N*,*N*,*N*′,*N*′-tetrakis(2-pyridylmethyl)ethylenediamine
WHO	World Health Organization
ZIP	Zrt-, Irt-like protein
Zn	zinc
ZnT	zinc transporter
