# Supplementary material for: A Guide to Human Zinc Absorption: General Overview and Recent Advances of In Vitro Intestinal Models
Source: Nutrients. 2020 Mar 13;12(3):762. doi: 10.3390/nu12030762 (PMC7146416; doi:10.3390/nu12030762)
Supplement: Supplementary file 1 [file nutrients-12-00762-s001.pdf]

Supplemental Material

A guide to human zinc absorption: general overview and recent advances of *in vitro* intestinal models

Maria Maares [1], Hajo Haase [1,2,*]

[1] Technische Universität Berlin, Chair of Food Chemistry and Toxicology, Germany

[2] TraceAge - DFG Research Unit on Interactions of essential trace elements in healthy and diseased elderly, Potsdam-Berlin-Jena, Germany

*Corresponding author:

Technische Universität Berlin, Chair of Food Chemistry and Toxicology, Straße des 17. Juni 135, 10623 Berlin, Germany Tel: +49 (0) 30 31472701
Fax: +49 (0) 30 31472823
E-Mail: Haase@TU-Berlin.de

Table 1. Zinc content of the human body.

Tissue	Weight (g) *	Ref.	Zinc Concentration (µg/g tissue wet weight)	Ref.	Total Zinc Content (g)	Proportion of Total Body Zinc * (%)
Eye	7.5	[1]	1.3	[2]	<0.01	<0.01
Liver	1,500	[3]	58	[4]	0.09	3.4
Heart	331	[5]	26.5	[6]	0.01	0.3
Brain	1,407	[3]	11	[6]	0.02	0.6
Lung	840	[3]	16	[6]	0.01	0.5
Kidneys	266	[3]	55	[4]	0.01	0.6
Intestine	2,100	[7]	15.5	[6]	0.03	1.3
Stomach	130	[8]	13.4	[6]	<0.01	0.1
Bone	9,458	[9]	100	[4]	0.95	36.7
Muscle	25,100	[10]	51	[4]	1.28	49.7
Blood plasma	3,437	[11]	1.25 [µg/mL]	[12]	<0.01	0.2
Whole Blood	5,509	[11]	6.81 [µg/mL]	[6]	0.04	1.5
Hair and nails	53.8	[9]	247	[13]	0.01	0.5
Skin	3,405.54	[9]	32	[4]	0.11	4.2
Spleen	139	[3]	14.7	[6]	<0.01	0.1
Pancreas	119	[14]	33.3	[15]	<0.01	0.2
					**Σ 2.6**	**100**

* calculated for a 60–70 kg adult, based on the respective body weight used as references for tissue weight.

Table 2. Recommended daily allowance for dietary zinc intake for selected life-stages.

WHO [16]				EFSA [17]				DGE [18]						
**Age, Sex**	**RNI (mg/d)**			**Age**		**PRI (mg/d)**		**Age**	**RDI (mg/d)**					
	**High** [a]	**Mod** [b]	**Low** [c]											
**7–12 mos**	0.8 [d]; 2.5 [e]	4.1	8.4	**7–11 mos**		2.9		**<4 mos**	1.5					
**1–3 yr**	2.4	4.1	8.3	**1–3 yr**		4.3		**4–12 mos**	2.5					
**4–6 yr**	2.9	4.8	9.6	**4–6**		5.5		**1–4 yr**	3.0					
**7–9 yr**	3.3	5.6	11.2	**7–10**		7.4		**4–7 yr**	4.0					
**10–18 yr, m**	5.1	8.6	17.1			**m**	**f**	**7–10 yr**	6.0					
**10–18 yr, f**	4.3	7.2	14.4	**11–14 yr**		9.4	9.4		**m**			**f**		
**19–65 yr, m**	4.2	7.0	14.0	**15–17 yr**		12.5	10.4	**Phytate**	Low [f]	Med [g]	High [h]	Low [f]	Med [g]	High [h]
**19–65 yr, f**	3.0	4.9	9.8	**Age**	**Phytate** (mg/d)			**10–13 yr**		9			8	
**> 65 yr, m**	4.2	7.0	14.0	**≥ 18 yr**	**300**	9.4	7.5	**13–15 yr**		12			10	
**> 65 yr, f**	3.0	4.9	9.8	**≥ 18 yr**	**600**	11.7	9.3	**15–19 yr**		14			11	
**Pregnancy**				**≥ 18 yr**	**900**	14.0	11.0	**≥19 yr**	11	14	16	7	8	10
1st TT	3.4	5.5	11.0	**≥ 18 yr**	**1200**	16.3	12.7	**Pregnancy**						
2nd TT	4.2	7.0	14.0					1st TT				7	9	11
3rd TT	6.0	10.0	20.0	**Pregnancy**			+1.6	2nd–3rd TT				9	11	13
**Lactation**				**Lactation**			+2.9	**Lactation**				11	13	14
0–3 mo	5.8	9.5	19.0											
3–6 mo	5.3	8.8	17.5											
6–12 mo	4.3	7.2	14.4											

BV, bioavailability; EFSA, European Food Safety Authority; DGE, German Society for Nutrition *(ger.: Deutsche Gesellschaft für Ernährung);* f, female; m, male; mos, months; PRI, population reference intake; RDI, recommended daily intake; RNI, recommended nutrient intake; TT, trimester; WHO, World Health Organization; yr, years; [a]High bioavailability (50%); [b]Moderate bioavailability (30%); [c]Low bioavailability (15%); [d]exclusively breastfed infants (bioavailability 80%); [e]not exclusively breastfed; [f] 300 mg phytate/d; [g] 660 mg phytate/d; [h] 990 mg phytate/d; Recommendations for adults from EFSA and DGE include different phytate levels using a trivariate model by Miller *et al.* [19] for assessing the relationship between dietary phytate, dietary zinc, and absorbed zinc.

.

Table 3. Application of human *in vitro* intestinal models to study zinc-dependent gene expression in enterocytes.

Cell Model	Incubation Parameter	Analysis	Main Outcome	Reference
Caco-2<br>Differentiation time:<br>14 d<br>2D	Recombinant expression of myc-tagged hZnT-5B in Caco-2 cells<br>Addition of $ZnCl_2$ to growth medium:<br>Stepwise increase from 20, 50 and 100 µM each for 7d	Recombinant transfection<br>Gene expression:<br>RT-PCR<br>Immunochemical staining	- highest expression of ZTL1 in mouse kidney, brain, duodenum and jejunum<br>- apical localization of hZTL1 at apical membrane of Caco-2<br>- hZTL1 (later named ZnT-5B) and MT expression increased in Caco-2-WT cells after prolonged zinc treatment	[20]
Caco-2<br>Cultivation time:<br>14 d<br>2D	Human study: 25 mg $ZnSO_4$/d<br>(placebo Na SO_4);<br>duration: 14 d<br><br>Caco-2: 100 µM or 200 µM $ZnCl_2$<br>(in DMEM + 10% FCS)<br>for 3 d	Gene expression:<br>RT-PCR<br>Protein quantification:<br>Immunocytochemistry	- mRNA and protein expression of ZnT-1, ZnT-5, ZnT-5, ZIP4 in enterocytes (biopsies of ileal mucosa) ↓<br>- *znt-1* ↓<br>- MT mRNA increased ↑<br>- mRNA and protein expression in Caco-2 cells was in agreement with human study<br>- localization of ZnT-5 at apical membrane of human enterocytes and Caco-2 cells	[21]
Caco-2<br>Cultivation time:<br>24 h<br>2D	0-100 µM $ZnCl_2$ (in serum-free DMEM)	Transient transfection of Caco-2 cells with pEGFP-ZnT5B	- ZnT-5 variant b is a bidirectional zinc transporter and can operate in an efflux mode, increasing cytoplasmic zinc concentration of Caco-2 cells<br>- upregulation of MT-2 indicates increase of intracellular zinc content in transfected Caco-2 cells	[22]
Caco-2<br>Cultivation time:<br>24 h, pre-confluent<br>2D	0-300 µM $ZnSO_4$<br>or 0-10 µM TPEN<br>(in n.a.)<br>for 6 or 12 h	Gene expression:<br>*q*PCR	- zinc-dependent mRNA expression of *mt-1*, *dmt-1*, *zip4* and *znt-1* regulates zinc homeostasis in Caco-2 cells<br>- *zip4* ↑ after zinc depletion with TPEN<br>- *mt-1* ↑ *and znt-1* with added zinc concentration	[23]

Caco-2 Differentiation time:<br>11-13 d<br>2D	Iron/zinc interaction<br>0-200 µM $ZnCl_2$ or $FeCl_3$, respectively, (in DMEM)<br>for 2h	Zinc uptake: radioactive zinc (^{65}Zn)	- iron uptake was inhibited dose-dependently by zinc<br>- iron increased cellular zinc uptake<br>- analysis suggests that iron and zinc transport by DMT-1 is not occurring simultaneously	[24]
Caco-2<br>Cultivation time:<br>14 d<br>2D	3-100 µM $ZnCl_2$ (in DMEM+ 10%FCS)<br>for 12 or 24 h	Transcriptomic study:<br>Micro-array<br>Gene expression:<br>*q*PCR	- zinc-regulated genes were analyzed with an micro-array<br>- identification of several genes which are regulated zinc-dependent (such as *mt-1h*, *mt-2a*, *mt-3*, *mtf-1*)	[25]
Caco-2<br>Cultivation time:<br>21d<br>3D Transwell<br>(comparison undifferentiated and differentiated cells)	100-800 µM $ZnCl_2$ (in DMEM + 5% FCS)<br>apical or basolateral incubation)<br>for 24 h	Gene expression:<br>*q*PCR	- influence of polarization and differentiation of Caco-2 cells on zinc tolerance<br>- mRNA expression of *znt-1* ↑, *znt-5*, *zip1*, *zip4*, *mt-1a* ↑, *mt-1x* ↑, *mt-2a* ↑ after exposure with higher zinc concentrations (100-800 µM; apical or basolateral, respectively)<br>- under physiologic zinc concentrations (apical: 100 µM; basolateral: 15 µM zinc) only *mt-1a* ↑	[26]
Caco-2 (1)<br><br>FHs 74 Int cells (2)<br>Cultivation time (1):<br>Undifferentiated (U)<br>(4 d)<br>Differentiated (D)<br>(12 d)<br>2D	50 µM $ZnSO_4$ (in serum free medium)<br>for 15 min	Zinc uptake: radioactive zinc (^{65}Zn)<br>Gene expression:<br>*q*PCR<br>Western Blot<br>Biotinylation of surface proteins	- role of zinc exposure on intestinal cells of varying maturity;<br>- zinc uptake in fetal intestinal cells and undifferentiated cells was higher than in differentiated cells<br>- ZnT-1 protein and *znt-1*, *znt-2* as well as *mt-1* ↑, *while zip4* ↑ in U and ↓ in D Caco-2 cells<br>- localization of ZIP4 and ZnT-1 at the plasma membrane of differentiated Caco-2 cells was significantly changed by zinc exposure	[27]
Caco-2<br>confluent cells<br>2D; 3D Transwell	0-100 µM $ZnSO_4$ (DMEM +10% FCS)<br>for 7 d	Zinc Uptake: total Zn<br>Western blot	- cellular zinc content increased concentration-dependent (100 µM: 0.4 µg mg^{-1} protein)	[28]

			- expression of TJ protein claudin-2 and tricellulin decreased with added zinc concentration<br>- TEER increased with added zinc concentration	
Caco-2<br>Cultivation time:<br>Undifferentiated: n.a.<br>Differentiated: 21d<br>2D; 3D Transwell<br>(EHS-coated matrix membrane)	100 µM $ZnCl_2$ (DMEM +10% FCS)<br>for 48 h	Transient transfection of Caco-2 cells with hZIP1<br>Gene expression:<br>*q*PCR<br>Zinc uptake: radioactive zinc (^{65}Zn)<br>Immunocytochemistry	- role of hZIP1 in intestinal epithelial cells<br>- hZIP1 tend to localize to the microvilli of Caco-2 cells during differentiation<br>- Caco-2 cells overexpressing hZIP1 accumulated intracellular zinc<br>- hZIP1 might act as a zinc sensing protein	[29]
Caco-2 (1)<br>IPEC-J2 (2)<br>Cultivation time (1):<br>Pre-confluent<br>(2-3 d)<br>Post-confluent<br>(19-21 d)<br>2D	0-200 µM $ZnSO_4$ (in DMEM +10% FCS)<br>for 6 h and 24 h	Zinc uptake: total Zn<br>Gene expression:<br>*q*PCR	- cellular zinc uptake increases significantly after incubating with 200 µM zinc for 24 h<br>- zinc incubation of post-confluent Caco-2 cells did not change *zip-4* and only showed a trend in *mt1a* and *znt-1* upregulation<br>- enterocyte zinc homeostasis is maintained by expression of these genes	[30]
Caco-2 (1)<br>IPEC-J2 (2)<br>Cultivation time (1):<br>21 d<br>3D Transwell	0-200 µM $ZnSO_4$ (apical or basolateral side, in DMEM + 10% FCS)<br>for 24 h	Gene expression:<br>*q*PCR	- *znt-1* and *mt* expression ↑ with higher added zinc concentrations basolaterally<br>- *zip4* expression did not change	[31]
Caco-2<br>Cultivation time:<br>24 h	3 or 150 µM zinc<br>(in serum free DMEM)<br>for 24 h	MTF-1 depletion by transient transfection with siRNA<br>MT-2a stable transfection<br>Transiently transfection with ZnT-5 promotor<br>Gene expression:<br>Microarray<br>*q*PCR	- zinc-dependent expression of MTF-1 dependent genes in MTF-1 depleted Caco-2 compared to CTR:<br>*znt-1*) ↓and *mt-1b*) ↓, *mt-1e*) ↓, *mt-1g*) ↓, *mt-1h*) ↓, *mt-1m*) ↓, *mt-2a*) ↓, *mt-1a* , *mt-2a* and *mt-x* did not change<br>- in MTF-1 depleted cells, zinc incubation changed mRNA expression of genes that are normally not affected by increased cellular zinc,	[32]

			indicating that MT and ZnT-1 are buffering their expression<br>- MT-2a overexpressed Caco-2 cells showed higher ZnT-5 promoter activity upon zinc uptake<br>- MTF-1 is controlling intracellular zinc homeostasis by regulating MT and ZnT-1

3D, three-dimensional; DMEM, Dulbecco's Modified Eagles Medium; EHS, Engelbreth-Holm-Swarm cells; (FCS, fetal calf serum; HBSS, Hank's Balanced Salt Solution; n.a., not available; PC, polycarbonate; TEER, transepithelial electrical resistance; TJ, tight junction; Zn, zinc.

Table 4. Application of *in vitro* Caco-2 monocultures to investigate the effect of dietary factors on zinc bioavailability.

Cell Model	Zinc Added	Food Component or Ligand	Quantification	Main Outcome	Reference
Caco-2 Cultivation time: 10-12 d 2D and 3D Transwell	$ZnSO_4$ $FeCl_3$ (apical: HEPES buffer, basolateral: DMEM + 15% FCS) for 1 h (uptake), 1-5 h (transport)	- Inositolphosphates (IP) (phytic acid): IP3, IP4, IP5, IP6	radioactive zinc and iron (^{65}Zn, ^{55}Fe)	- inhibition of iron and zinc transport by phytate in Caco-2<br>- reduction of zinc uptake and transport rate correlated with level phosphorylation (IP3 to IP6)<br>- cellular uptake was analyzed in 2D, transport with 3D transwell	[33]
Caco-2 Cultivation time: 15-18 d 2D	40.22 μM $ZnCl_2$, 88.24 μM $FeCl_3$ or 823.53 μM $CaCl_2$ respectively (in uptake buffer)	- infant formulas: adapted (milk based) and soy-based<br>- *in vitro* digestion model	total zinc	- lower zinc uptake von soy-based than from milk-based infant formulas<br>- cellular zinc uptake solely observed from digested infant formulas and not from liquid metal solutions	[34]
Caco-2 Cultivation time: 19 – 21 d 3D Transwell (PE membrane)	sample [c] (apical: soluble mineral fraction, basolateral: HBSS buffer) for 2 h	- raw legumes: white beans, chickpeas, lentils<br>- effect on cooking of lentils<br>- *in vitro* digestion model	total zinc	- chickpeas yielded the highest amount of transported zinc<br>- cooking process negatively affected the mineral content of lentils and the soluble zinc fraction decreased	[35]
Caco-2 Cultivation time: 21 d 3D Transwell (PES membrane)	sample [c] (apical: soluble mineral fraction; basolateral: HBSS buffer) for 2 h	- school meals<br>- *in vitro* digestion model	total zinc	- iron, copper, zinc and calcium uptake and transport was analyzed<br>- protein content of meals had no influence on zinc uptake<br>- negative mineral interaction of iron and zinc:	[36]

				soluble iron decreased and transported zinc; soluble zinc and iron retention	
Caco-2<br>Cultivation time: 14-12 d<br>2D	25 µM $^{65}ZnCl_2$<br>(in MEM)<br>for 3 h	- phytic acid, tannic acid, tartaric acid, polyphenols (from tea extract and grape juice), wheat, arginine, methionine, histidine<br>- molar ratio: zinc/dietary ligands (1:1; 1:5; 1:10)<br>- *in vitro* digestion model (use of dialysis membrane for incubation of cells with digested samples)	radioactive zinc (^{65}Zn)	- zinc depletion with TPEN increased zinc uptake, but zinc repletion did not affect uptake<br>- zinc uptake in Caco-2 cells shows a saturable and non-saturable component depending on added zinc concentration<br>- tannic acid (1:50) enhanced zinc uptake from wheat- and rice-food-matrix<br>- histidine, phytate, tartaric acid (1:1) and methionine (1:10) resulted in decreased zinc uptake relative to control cells	[37]
Caco-2<br>Cultivation time: 14-21 d<br>3D Transwell (PE membrane)	sample [c]<br>(in salt buffer)<br>for 2 h	- influence of caseinophosphopeptides (CPPs) and milk on zinc uptake from fruit beverages<br>- *in vitro* digestion model	total zinc	- zinc retention, transport and uptake was higher for milk-containing fruit beverages than for CPPs-based fruit beverages	[38]
Caco-2<br>Cultivation time:<br>21 d<br>3D Transwell (PC membrane)	sample [c]<br>(apical: HEPES,MES, glucose, basolateral: HBSS)<br>for 3 h	- cereals and dephytinized cereals (phytase)<br>- *in vitro* digestion model	total zinc	- effect of dephytinization on zinc, iron and calcium bioavailability in Caco-2 cells<br>- zinc and iron solubility and fractional zinc and iron absorption increased after dephytinization of cereals	[39]
Caco-2<br>Cultivation time: 11-13 d<br>2D	50 µM $^{65}ZnCl_2$<br>Iron-zinc interactions:<br>Zn:Fe (1:1)	- ascorbic acid (1 mM) and phytic acid, tannic acid, tartaric acid, cysteine, histidine, methionine (each 500 µM)	radioactive zinc (^{65}Zn)	- ascorbic acid, tartaric acid and tannic acid increased zinc uptake	[40]

	(in DMEM)<br>for 2 h			- phytic acid and histidine decreased cellular zinc uptake<br>- increase of iron uptake in presence of methionine, increased also zinc uptake<br>- without added ligands, zinc inhibited iron uptake into Caco-2<br>- ligands can modulate iron : zinc-interaction	
Caco-2<br>Cultivation time: 12-14 d<br>2D	25 µM $^{65}ZnCl_2$<br>(in MEM)<br>for 3 h	- polyphenol-rich beverages: red wine, green tea, red grape juice<br>- tannic acid, quercetin, gallic acid, caffeic acid (each 250µM)<br>- *in vitro* digestion model (including a rice matrix)	radioactive zinc (^{65}Zn)	- polyphenol-rich beverages increased cellular zinc uptake from digested rice matrix<br>- tannic acid and quercetin enhanced zinc uptake	[41]
Caco-2<br>Cultivation time: 21-28 d<br>3D Transwell (PET-HD membrane)	50 µM zinc<br>(in HEPES buffer)<br>for 1 h	- water soluble vitamins: folic acid, nicotinic acid, ascorbic acid, riboflavin, thiamine, pyridoxine<br>- effect of oxidative species on vitamin-dependent zinc uptake was analyzed<br>- phytic acid and histidine	total zinc	- zinc transport was slightly enhanced by nicotinic acid and slightly decreased by thiamine, riboflavin, and pyridoxine<br>- phytic acid significantly decreased zinc uptake compared to control cells, where histidine resulted in a slight increase of zinc uptake	[42]
Caco-2<br>Cultivation time:<br>21 d<br>3D Transwell (PC membrane)	sample [c]<br>(in apical and basolateral HBSS)<br>for 1 h	- samples from each stage of processing: wheat flour, whole wheat flour; fermented and final product: white bread, whole wheat bread, muffin<br>- *in vitro* digestion model	total zinc	- effect of 'processing' of baking products on bioavailability of calcium, iron and zinc in Caco-2 cells<br>- no differences in zinc uptake from fermented dough and after baking	[43]

Caco-2 Cultivation time: 21 d 2D	0 - 25 µM $ZnCl_2$ (in minimum essential medium) for 24 h	- varying zinc : phytic acid-ratios<br>- *in vitro* digestion model (red beans, fish samples)	stable zinc isotope (n.a.) MT quantification [a]	- MT formation was investigated as a proxy for zinc uptake<br>- phytic acid significantly decreased zinc uptake and MT formation for Zn : phytic acid ratios < 1:5	[44]
Caco-2 Cultivation time: 17 d 3D Transwell (collagen coated)	10 µM $^{65}ZnCl_2$ (apical: HBSS buffer, basolateral: DMEM) for 1-3 h	- bioactive dietary polyphenols: epigallocatechin-3-gallate (EGCG), green tea extract (GT), and grape seed extract (GSE) (each 46 mg/L)<br>- phytate (100 µM)	radioactive zinc (^{65}Zn)	- GSE decreased zinc absorption by inhibiting cellular zinc uptake, similar to phytate<br>- EGCG and GT did not reduce zinc absorption	[45]
Caco-2 Cultivation time: 14 d 2D	sample [c] (in medium) for 6 h	- different rice varieties and zinc biofortified rice (polished or parboiled samples)<br>- *in vitro* digestion model	radioactive zinc (^{65}Zn)	- comparison of zinc uptake from biofortified rice with *in vivo* rat pups<br>- biofortified rice yielded significant higher net absorption *in vitro* and *in vivo*<br>- net absorption was smaller in Caco-2 but showed the same correlation between different rice samples	[46]
Caco-2 Cultivation time: 13 d 2D	sample [c] for 2 h followed by additional incubation for 10 h	- biofortified wheat (low-phytate mutants with varying zinc content)<br>- *in vitro* digestion model	Reporter gene assay [b]	- positive and negative correlations for zinc bioavailability dependent on zinc : phytate-ratios	[47]
Caco-2 Cultivation time: n.a. 2D	sample [c] (in MEM + 3% FCS) for 6 h	- reduction of phytate content in sorghum (genetic modification)<br>- *in vitro* digestion model	radioactive zinc and iron (^{65}Zn, ^{59}Fe)	- comparison of fractional absorption in Caco-2 and *in vivo* suckling rat pup model<br>- phytate reduction significantly increased zinc	[48]

				bioavailability in Caco-2 cells comparable to *in vivo* analysis	
Caco-2 Transwell (PE) Cultivation time: 21 d 3D Transwell (PE membrane)	250 µM $ZnSO_4$ (in DPBS) for 2 h	- GPAGPHGPPG peptide (derived from Alaska pollock)	total Zn	- influence of GPAGPHGPPG peptide on zinc, iron and calcium transport<br>- GPAGPHGPPG peptide significantly increased mineral transport.	[49]
Caco-2 Cultivation time: 10 d 2D	50 µM $ZnCl_2$ (in PBS) for 30 min	- amino acids (AAs): glutamate (Glu), lysine (Lys), methionine (Met)<br>- ZnAAs complexes: ZnGlu, ZnMet, ZnLys	Free zinc (Fluorescent zinc sensor Zinpyr-1)	- ZnAAs are probably absorbed by AAs transporters<br>- zinc uptake into Caco-2 cells is not enhanced by ZnAAS complexes<br>- results suggest that ZnAAs represent a more efficient war for zinc supplementation that zinc salts; especially for AE patients	[50]

3D, three-dimensional; AAs, amino acids BSA, bovine serum albumin; DMEM, Dulbecco's Modified Eagles Medium; FCS, fetal calf serum; HBSS, Hank's Balanced Salt Solution; HD, high density; HEPES, 4-(2-hydroxyethyl)-1-piperazineethanesulfonic acid;; IP, inositolphosphate; MEM, minimum essential medium; n.a., not available; PC, polycarbonate; PE, polyethylene; PES, polyester; Zn, zinc; [a] MT formation was analyzed using a cadmium/hemoglobin assay; [b] reporter gene assay based on the metal response element (MRE)-binding transcription factor-1 (MTF-1) and MRE luciferase, [c] mineral bioavailability from the sample solely was examined; no extra zinc added.

Abbreviations:

2D	two-dimensional
3D	three-dimensional
DGE	German Society for Nutrition; ger. *Deutsche Gesellschaft für Ernährung*
DMEM	Dulbecco's Modified Eagles Medium
DMT-1	divalent metal transporter
EFSA	European Food Safety Authority
EHS	Engelbreth-Holm-Swarm cells
FAAS	flame atomic absorption spectrometry
FCS	fetal calf serum
HD	high density
HBSS	Hanks' Balanced Salt Solution
HEPES	4-(2-hydroxyethyl)-1-piperazineethanesulfonic acid
IP	inositolphosphate
KHB	Krebs-Henseleit buffer
LMW	low molecular weight
mRNA	messenger ribonucleic acid
MEM	minimum essential medium
MT	metallothionein
MTF-1	metal regulatory transcription factor 1
PBMC	peripheral blood mononuclear cells
PBS	phosphate buffered saline
PC	polycarbonate
PE	polyethylene
PES	polyester
PET	photo-induced electron transfer
*q*PCR	quantitative real time polymerase chain reaction (PCR)
TEER	transepithelial electrical resistance
TJ	tight junction
TPEN	N,N,N′,N′-tetrakis(2-pyridylmethyl)ethylenediamine
WHO	World Health Organization
ZIP	Zrt-, Irt-like protein
Zn	zinc
ZnT	zinc transporter

Supplementary References:

1. Sachsenweger, M. *Augenheilkunde*. 2003; Vol. 2.
2. Karcioglu, Z.A. Zinc in the eye. *Survey of Ophthalmology* **1982**, *27*, 114-122.
3. Molina, D.K.; DiMaio, V.J. Normal organ weights in men: Part ii-the brain, lungs, liver, spleen, and kidneys. *Am J Forensic Med Pathol* **2012**, *33*, 368-372.
4. Jackson, M.J. Physiology of zinc: General aspects. In *Zinc in human biology*, Mills, C.F., Ed. Springer London: London, 1989; pp 1-14.
5. Molina, D.K.; DiMaio, V.J. Normal organ weights in men: Part i-the heart. *Am J Forensic Med Pathol* **2012**, *33*, 362-367.
6. Lech, T.; Sadlik, J.K. Zinc in postmortem body tissues and fluids. *Biological trace element research* **2010**, *142*, 11-17.
7. Yamanaka, H.; Nakajima, M.; Katoh, M.; Yokoi, T. Glucuronidation of thyroxine in human liver, jejunum, and kidney microsomes. *Drug Metabolism and Disposition* **2007**, *35*, 1642.
8. Boyd, R. Xii. Tables of the weights of the human body and internal organs in the sane and insane of both sexes at various ages, arranged from 2614 post-mortem examinations. *Philosophical Transactions of the Royal Society of London* **1861**, *151*, 241-262.
9. Forbes, R.M.; Cooper, A.R.; Mitchell, H.H. The composition of the adult human body as determined by chemical analysis. *J Biol Chem* **1953**, *203*, 359-366.
10. Clarys, J.P.; Martin, A.D.; Drinkwater, D.T. Gross tissue weights in the human body by cadaver dissection. *Human Biology* **1984**, *56*, 459-473.
11. Yiengst, M.J.; Shock, N.W. Blood and plasma volume in adult males. *Journal of Applied Physiology* **1962**, *17*, 195-198.
12. Folin, M.; Contiero, E.; Maria Vaselli, G. Zinc content of normal human serum and its correlation with some hematic parameters. *Biometals* **1994**, *7*, 75-79.
13. Wilhelm, M.; Hafner, D.; Lombeck, I.; Ohnesorge, F.K. Monitoring of cadmium, copper, lead and zinc status in young children using toenails: Comparison with scalp hair. *The Science of the total environment* **1991**, *103*, 199-207.
14. Scott, D.A.; Fisher, A.M. The insulin and the zinc content of normal and diabetic pancreas. *The Journal of clinical investigation* **1938**, *17*, 725-728.
15. Rahil-Khazen, R.; Bolann, B.J.; Myking, A.; Ulvik, R.J. Multi-element analysis of trace element levels in human autopsy tissues by using inductively coupled atomic emission spectrometry technique (icp-aes). *Journal of Trace Elements in Medicine and Biology* **2002**, *16*, 15-25.
16. World Health Organization / Food and Agricultural Organization. *Vitamin and mineral requirements in human nutrition*. 2 ed.; World Health Organization: Geneva, Switzerland, 2004.
17. EFSA Panel on Dietetic Products, N.a.A.N. Scientific opinion on dietary reference values for zinc. *EFSA Journal 2014* **2014**, *12*.
18. Deutsche Gesellschaft für Ernährung; Österreichische Gesellschaft für Ernährung; Schweizerische Gesellschaft für Ernährung. *Referenzwerte für die nährstoffzufuhr*. 2 ed.; Deutsche Gesellschaft für Ernährung e. V.: Bonn, Germany, 2019; Vol. 2.
19. Miller, L.V.; Krebs, N.F.; Hambidge, K.M. A mathematical model of zinc absorption in humans as a function of dietary zinc and phytate. *The Journal of nutrition* **2007**, *137*, 135-141.
20. Cragg, R.A.; Christie, G.R.; Phillips, S.R.; Russi, R.M.; Kury, S.; Mathers, J.C.; Taylor, P.M.; Ford, D. A novel zinc-regulated human zinc transporter, hztl1, is localized to the enterocyte apical membrane. *The Journal of Biological Chemistry* **2002**, *277*, 22789-22797.
21. Cragg, R.A.; Phillips, S.R.; Piper, J.M.; Varma, J.S.; Campbell, F.C.; Mathers, J.C.; Ford, D. Homeostatic regulation of zinc transporters in the human small intestine by dietary zinc supplementation. *Gut* **2005**, *54*, 469-478.
22. Valentine, R.A.; Jackson, K.A.; Christie, G.R.; Mathers, J.C.; Taylor, P.M.; Ford, D. Znt5 variant b is a bidirectional zinc transporter and mediates zinc uptake in human intestinal caco-2 cells. *The Journal of Biological Chemistry* **2007**, *282*, 14389-14393.
23. Shen, H.; Qin, H.; Guo, J. Cooperation of metallothionein and zinc transporters for regulating zinc homeostasis in human intestinal caco-2 cells. *Nutrition Research* **2008**, *28*, 406-413.

24. Pieri, M.; Christian, H.C.; Wilkins, R.J.; Boyd, C.A.; Meredith, D. The apical (hpept1) and basolateral peptide transport systems of caco-2 cells are regulated by amp-activated protein kinase. *American Journal of Physiology - Gastrointestinal and liver physiology* **2010**, *299*, G136-143.
25. Jackson, K.A.; Valentine, R.A.; McKay, J.A.; Swan, D.C.; Mathers, J.C.; Ford, D. Analysis of differential gene-regulatory responses to zinc in human intestinal and placental cell lines. *The British journal of nutrition* **2009**, *101*, 1474-1483.
26. Zemann, N.; Zemann, A.; Klein, P.; Elmadfa, I.; Huettinger, M. Differentiation- and polarization-dependent zinc tolerance in caco-2 cells. *European journal of nutrition* **2011**, *50*, 379-386.
27. Jou, M.Y.; Philipps, A.F.; Kelleher, S.L.; Lonnerdal, B. Effects of zinc exposure on zinc transporter expression in human intestinal cells of varying maturity. *Journal of pediatric gastroenterology and nutrition* **2010**, *50*, 587-595.
28. Wang, X.; Valenzano, M.C.; Mercado, J.M.; Zurbach, E.P.; Mullin, J.M. Zinc supplementation modifies tight junctions and alters barrier function of caco-2 human intestinal epithelial layers. *Digestive Diseases and Sciences* **2012**, *58*, 77-87.
29. Michalczyk, A.A.; Ackland, M.L. Hzip1 (hslc39a1) regulates zinc homoeostasis in gut epithelial cells. *Genes & nutrition* **2013**, *8*, 475-486.
30. Gefeller, E.M.; Bondzio, A.; Aschenbach, J.R.; Martens, H.; Einspanier, R.; Scharfen, F.; Zentek, J.; Pieper, R.; Lodemann, U. Regulation of intracellular zn homeostasis in two intestinal epithelial cell models at various maturation time points. *The Journal of Physiological Sciences* **2015**, *65*, 317-328.
31. Lodemann, U.; Gefeller, E.M.; Aschenbach, J.R.; Martens, H.; Einspanier, R.; Bondzio, A. Dose effects of apical versus basolateral zinc supplementation on epithelial resistance, viability, and metallothionein expression in two intestinal epithelial cell lines. *Journal of biochemical and molecular toxicology* **2015**.
32. Hardyman, J.E.; Tyson, J.; Jackson, K.A.; Aldridge, C.; Cockell, S.J.; Wakeling, L.A.; Valentine, R.A.; Ford, D. Zinc sensing by metal-responsive transcription factor 1 (mtf1) controls metallothionein and znt1 expression to buffer the sensitivity of the transcriptome response to zinc. *Metallomics : integrated biometal science* **2016**, *8*, 337-343.
33. Han, O.; Failla, M.L.; Hill, A.D.; Morris, E.R.; Smith, J.C., Jr. Inositol phosphates inhibit uptake and transport of iron and zinc by a human intestinal cell line. *The Journal of nutrition* **1994**, *124*, 580-587.
34. Jovani, M.; Barbera, R.; Farre, R.; Aguilera, E.M.d. Calcium, iron, and zinc uptake from digests of infant formulas by caco-2 cells. *Journal of Agricultural and Food Chemistry* **2001**, *49*, 3480−3485.
35. Viadel, B.; Barberá, R.; Farré, R. Uptake and retention of calcium, iron, and zinc from raw legumes and the effect of cooking on lentils in caco-2 cells. *Nutrition Research* **2006**, *26*, 591-596.
36. Cámara, F.; Barberá, R.; Amaro, M.A.; Farré, R. Calcium, iron, zinc and copper transport and uptake by caco-2 cells in school meals: Influence of protein and mineral interactions. *Food Chemistry* **2007**, *100*, 1085-1092.
37. Sreenivasulu, K.; Raghu, P.; Ravinder, P.; Nair, K.M. Effect of dietary ligands and food matrices on zinc uptake in caco-2 cells: Implications in assessing zinc bioavailability. *Journal of Agricultural and Food Chemistry* **2008**, *56*, 10967-10972.
38. García-Nebot, M.J.; Alegría, A.; Barberá, R.; Clemente, G.; Romero, F. Does the addition of caseinophosphopeptides or milk improve zinc in vitro bioavailability in fruit beverages? *Food Research International* **2009**, *42*, 1475-1482.
39. Frontela, C. Effect of dephytinization on bioavailability of iron, calcium and zinc from infant cereals assessed in the caco-2 cell model. *World Journal of Gastroenterology* **2009**, *15*, 1977.
40. Iyengar, V.; Pullakhandam, R.; Nair, K.M. Dietary ligands as determinants of iron-zinc interactions at the absorptive enterocyte. *Journal of Food Science* **2010**, *75*, H260-264.
41. Sreenivasulu, K.; Raghu, P.; Nair, K.M. Polyphenol-rich beverages enhance zinc uptake and metallothionein expression in caco-2 cells. *Journal of Food Science* **2010**, *75*, H123-128.
42. Tupe, R.S.; Agte, V.V. Effect of water soluble vitamins on zn transport of caco-2 cells and their implications under oxidative stress conditions. *European journal of nutrition* **2009**, *49*, 53-61.
43. Frontela, C.; Ros, G.; Martínez, C. Phytic acid content and "in vitro" iron, calcium and zinc bioavailability in bakery products: The effect of processing. *Journal of Cereal Science* **2011**, *54*, 173-179.
44. Cheng, Z.; Tako, E.; Yeung, A.; Welch, R.M.; Glahn, R.P. Evaluation of metallothionein formation as a proxy for zinc absorption in an in vitro digestion/caco-2 cell culture model. *Food & function* **2012**, *3*, 732-736.

45. Kim, E.-Y.; Pai, T.-K.; Han, O. Effect of bioactive dietary polyphenols on zinc transport across the intestinal caco-2 cell monolayers. *Journal of Agricultural and Food Chemistry* **2011**, *59*, 3606-3612.
46. Jou, M.-Y.; Du, X.; Hotz, C.; Lönnerdal, B. Biofortification of rice with zinc: Assessment of the relative bioavailability of zinc in a caco-2 cell model and suckling rat pups. *Journal of Agricultural and Food Chemistry* **2012**, *60*, 3650-3657.
47. Salunke, R.; Rawat, N.; Tiwari, V.K.; Neelam, K.; Randhawa, G.S.; Dhaliwal, H.S.; Roy, P. Determination of bioavailable-zinc from biofortified wheat using a coupled in vitro digestion/caco-2 reporter-gene based assay. *Journal of Food Composition and Analysis* **2012**, *25*, 149-159.
48. Kruger, J.; Taylor, J.R.N.; Du, X.; De Moura, F.F.; Lönnerdal, B.; Oelofse, A. Effect of phytate reduction of sorghum, through genetic modification, on iron and zinc availability as assessed by an in vitro dialysability bioaccessibility assay, caco-2 cell uptake assay, and suckling rat pup absorption model. *Food Chemistry* **2013**, *141*, 1019-1025.
49. Chen, Q.; Guo, L.; Du, F.; Chen, T.; Hou, H.; Li, B. The chelating peptide (gpagphgppg) derived from alaska pollock skin enhances calcium, zinc and iron transport in caco-2 cells. *International Journal of Food Science & Technology* **2017**, *52*, 1283-1290.
50. Sauer, A.K.; Pfaender, S.; Hagmeyer, S.; Tarana, L.; Mattes, A.K.; Briel, F.; Kury, S.; Boeckers, T.M.; Grabrucker, A.M. Characterization of zinc amino acid complexes for zinc delivery in vitro using caco-2 cells and enterocytes from hipsc. *BioMetals* **2017**, *30*, 643-661.
